# Carbon isotope discrimination as a key physiological trait to phenotype drought/heat resistance of future climate-resilient German winter wheat compared with relative leaf water content and canopy temperature

**DOI:** 10.3389/fpls.2022.1043458

**Published:** 2022-11-30

**Authors:** Karolin Kunz, Yuncai Hu, Urs Schmidhalter

**Affiliations:** School of Life Sciences, Technical University of Munich, Freising, Germany

**Keywords:** heat/drought tolerance, climate resilience, Eastern Europe, hybrid, leaf water content (RLWC), plant canopy temperature (CT), carbon isotope discrimination (CID), plant phenotyping

## Abstract

Climate change is expected to influence crop growth through frequent drought and heat extremes, and thus, drought and heat tolerance are of increasing importance as major breeding goals for cereal crops in Central Europe. Plant physiological water status traits are suitable for phenotyping plant drought/heat tolerance. The objective of this study was to determine whether relative leaf water content (RLWC), plant canopy temperature (CT), and carbon isotope discrimination (CID) are suitable for phenotyping the drought/heat resistance of German winter wheat for future climate resilience. Therefore, a comprehensive field evaluation was conducted under drier and warmer conditions in Moldova using a space-for-time approach for twenty winter wheat varieties from Germany and compared to twenty regionally adapted varieties from Eastern Europe. Among the physiological traits RLWC, CT, and CID, the heritability of RLWC showed the lowest values regardless of year or variety origin, and there was no significant correlation between RLWC and grain yield regardless of the year, suggesting that RLWC did not seem to be a useful trait for distinguishing origins or varieties under continental field conditions. Although the heritability of CT demonstrated high values, the results showed surprisingly low and nonsignificant correlations between CT and grain yield; this may have been due to a confounding effect of increased soil temperature in the investigated dark Chernozem soil. In contrast, the heritability of CID in leaves and grain was high, and there were significant correlations between grain yield and CID, suggesting that CID is a reliable indirect physiological trait for phenotyping drought/heat resistance for future climate resilience in German wheat.

## Introduction

Climate change that leads to frequent drought and heat extremes is a crucial problem limiting agricultural production. It is generally accepted that environmental changes will lead to increases in temperature and extended drought periods worldwide by 2050 (Powell et al., 2012). In Germany, early-season drought events have also been occurring more frequently in the recent past (Herbst and Frühauf, 2018; Bönecke et al., 2020; Kunzel et al., 2021; Marszalek et al., 2022). Precipitation patterns are expected to be altered considerably, with 20% less precipitation predicted in most parts of Germany during summer. Furthermore, studies show that high temperatures and drought in Europe, caused by global warming, frequently occur around the anthesis and grain-filling stage of wheat (Semenov et al., 2009; Trnka et al., 2011), at which time wheat is particularly sensitive to extreme heat and drought conditions (Porter and Gawith, 1999; Wahid et al., 2012; Alghabari et al., 2014; Prasad and Djanaguiraman, 2014). Therefore, drought and heat tolerance are becoming increasingly important major cereal crop breeding goals in Central Europe.

Drought/heat tolerance mechanisms involve a complex interaction among various traits. As plant physiology can provide new tools to understand the complex network of drought/heat-related traits that are useful for improving the phenotyping efficiency, physiological traits subjected to a combination of these two stresses have revealed that the stress combination has several unique aspects, such as combining high respiration with low photosynthesis, closed stomata and high leaf temperatures and a decreased relative leaf water content (Mittler, 2006). Drought and heat negatively affect the relative leaf water content (RLWC) in many field crops (Farooq et al., 2012; Becker and Schmidhalter, 2017; Rischbeck et al., 2017). The ability to maintain high levels of RLWC during growth helps to reduce heat stress in the plant tissue, decreases chlorophyll degradation, and extends the time required to carry out photosynthesis (Thomas and Howarth, 2000; Borrell et al., 2001; Park et al., 2007; Prasad et al., 2008; Rischbeck et al., 2014). The canopy temperature (CT), as a surrogate of stomatal conductance, has been related to crop water use and yield formation. It has been successfully applied to estimate the grain yield (Elsayed et al., 2015; Becker and Schmidhalter, 2017; Elsayed et al., 2017), plant water status, and plant drought tolerance (Gomez-Candon et al., 2016; Bellvert et al., 2016; Rischbeck et al., 2017). The CT was robustly associated with wheat’s water status and stomatal conductance (Blum et al., 1989; Amani et al., 1996). Low wheat CT during the grain-filling stage has been associated with a 30% increase in yield and increased water uptake by relatively deep roots (Lopes and Reynolds, 2010). The CT can be assessed nondestructively, and regular CT assessments during the breeding process provide great potential for the indirect selection of varieties with optimized rooting behavior. Thus, CT has been considered an important trait to select for adapted genotypes and is used for high-throughput phenotyping (Rischbeck et al., 2016). Under drought stress, carbon isotope discrimination (CID) is another good predictor of stomatal conductance (Condon et al., 2002) and water use efficiency (WUE) (Tambussi et al., 2007). Several studies on wheat have shown a close correlation between CID and the final grain yield under drought conditions. High genetic variation in grain CID has been reported with high heritability (Merah et al., 2001; Becker and Schmidhalter, 2017), indicating that CID is an important breeding target for improving the efficiency of water usage and yield under drought stress. The characterization of drought- and heat-stressed German and East European wheat cultivars is required to determine whether RLWC, CT, and CID can serve as indirect selection criteria to improve grain yields under drought/heat conditions.

To characterize drought- and heat-stressed German wheat cultivars facing future climate changes, there is a need to verify the response of wheat varieties from Central Europe by simulating anticipated or future climate conditions using environments where aggravated drought and conditions are already prevalent, such as in the continental regions of Eastern Europe. Since the latitude in central Moldova (e.g., Bălți 47° 46’ N, 27° 56’ E) is similar to that of southeastern Germany (e.g., Freising 48° 24’ N, 11° 44’ E) but the climate is drier and hotter, the conditions in Moldova may allow the anticipation of future drought and heat stress scenarios that are highly likely to occur in Germany. Field experiments under the rainfed conditions in the relatively dry, warm climate zone in Moldova present the novelty of this study and will allow for conclusive remarks on the stress tolerance of German wheat varieties. Furthermore, the tolerance of wheat to abiotic stresses is genetically determined and depends on the breeding history and the origin of the varieties (Gutierrez et al., 2010; Ratajczak and Górny, 2012; Barlow et al., 2015; Cao et al., 2015; Mäkinen et al., 2018; Mandea and Săulescu, 2018; Schittenhelm et al., 2019). Modern cultivars from Central Europe have high yield potential and are well adapted to optimal growing conditions with sufficient water availability (Ceccarelli et al., 1991; Mäkinen et al., 2018), while studies also show greater yield reductions under stress conditions (Dodig et al., 2012; Abu-Zaitoun et al., 2018; Mäkinen et al., 2018). Thus, well-adapted wheat varieties from hotter and drier areas could be used as important genetic sources for breeding programs in Western Europe (Schittenhelm et al., 2019) and could expand the genetic range of modern wheat varieties to achieve a higher degree of abiotic stress tolerance (Yadav, 2008; Abu-Zaitoun et al., 2018). Testing genetic resources from better adapted Eastern European wheat varieties under abiotic stress can help identify the drought/heat tolerance-related physiological traits of plants. In addition, identifying the physiological traits of Eastern European wheat varieties will play an important role in understanding their drought/heat stress tolerance and grant access to an important genetic source for improving the adaptation of German wheat to climate change.

Therefore, the main objective of this study was to determine reliable physiological traits (RLWC, CT, and CID) associated with heat and drought stress tolerance. Three-year field experiments involving twenty German wheat varieties were performed in northern Moldova and compared to twenty regionally adapted cultivars originating from Ukraine, Romania, Hungary, and Moldova. The present-day continental climate in Moldova provides a platform for natural stress conditions and allows the testing of wheat varieties in a realistic environment, as the frequent hot and dry periods that occur in Moldova during summer resemble Germany’s expected future climate scenarios.

## Materials and methods

### Plant materials, experimental design, and plant growth

Three-year field experiments were performed from 2017 to 2019 at the Selectia Research Institute of Field Crops in Bălți (47° 46’ N, 27° 56’ Z, 85 masl) in the Republic of Moldova, located at a comparable latitude with similar solar radiation conditions to reference sites in Germany where the investigated cultivars are normally grown. Regarding the climate conditions in Bălți, the average air temperature is higher in this region than in southern Germany during summer and autumn (April-October). Bălți also has considerably lower precipitation in most months. This allows us to evaluate the impacts of higher temperatures and decreased rainfall in anticipation of future climate changes.

The soil type in the experimental area is described as Chernozem, and the predominant soil texture is clay loam with a mean clay content of ca. 45%, with 4-5% sand and 50-51% silt (Boincean and Dent, 2019). The available soil water content down to 100 cm soil depth is 14%, considered moderately high and compares well to Tertiary hill soils in Southern Germany.

Forty varieties of winter wheat (*Triticum aestivum* L.) were grown, among which 20 cultivars were from Eastern European countries (Bulgaria, Moldova, Romania, and Ukraine) and 16 lines and four hybrids were from Germany. A list of the varieties can be found in **Table 1**.

**Table 1:** Wheat varieties used in the field experiments and their country of origin BG, Bulgaria; MD, Republic of Moldova; RO, Romania; UA, Ukraine; GER, Germany.

Variety	Origin	Breed	Variety	Origin	Breed
Acord	MD	Line	Akteur	Ger	Line
Amor	MD	Line	Anapolis	Ger	Line
Clasic	MD	Line	Apertus	Ger	Line
FGmut 293	RO	Line	Colonia	Ger	Line
Kuialnik	UA	Line	Discus	Ger	Line
Meleag	MD	Line	Elixer	Ger	Line
Numitor	MD	Line	Genius	Ger	Line
Pajura	RO	Line	Impression	Ger	Line
Rowina	RO	Line	JB Asano	Ger	Line
Savant	MD	Line	Kerubino	Ger	Line
Semnal	RO	Line	Kometus	Ger	Line
Slava	BG	Line	Manager	Ger	Line
Talisman	MD	Line	Mulan	Ger	Line
Transitor	RO	Line	Patras	Ger	Line
Ujinoc	UA	Line	Rumor	Ger	Line
Unitar	RO	Line	Tobak	Ger	Line
Ursita	RO	Line	Hybery	Ger	Hybrid
Zagrava	UA	Line	Hybred	Ger	Hybrid
Zisk	UA	Line	Hyfi	Ger	Hybrid
Zolotocolosa	UA	Line	Hystar*	Ger	Hybrid

*Hystar was used in 2017 and 2018; Hylux replaced Hystar in 2019.

**Figure 1:**
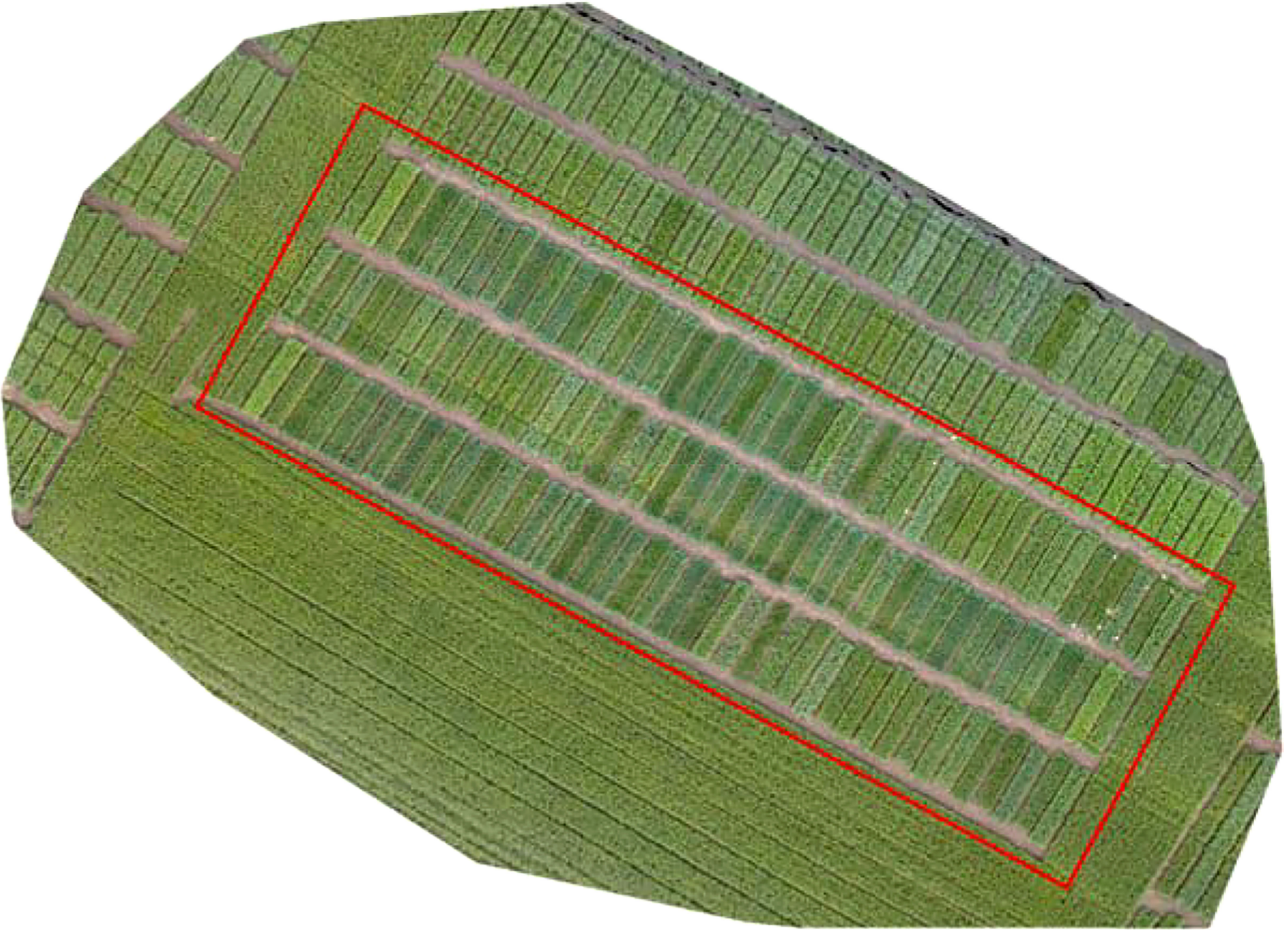
Aerial view of the experimental plots in May 2017.

The experiments were performed as a complete block design with three replicates. The plot size was 5×1 m, with a 12 cm row distance. **Figure 1** shows an aerial view of the experiments. The experimental site was part of a crop rotation experiment; the crop preceding winter wheat was alfalfa (*Medicago sativa* L.) in all years. Sowing with 500 grains m^-2^ took place on October 7, 2016, October 3, 2017, and October 8, 2018. Grains were harvested on July 11, 2017, July 2, 2018, and July 7, 2019. In the 2018/2019 season, the number of plots was doubled. Half served as rainfed abiotic stress treatments as in previous years, and the other half was irrigated. In the irrigated treatments, 60 liters of water per square meter were applied immediately after sowing.

Zadoks’ growth stages (Zadoks et al., 1974) were assessed from May until harvest. The plots were harvested with a plot combine harvester. After harvest, the yield of each plot was determined.

### Relative leaf water content (RLWC) and carbon isotope discrimination (CID)

Relative leaf water content (RLWC) was determined at Z71 (early milk or kernel water ripe) and Z79 (late milk) stages and carbon isotope discrimination (CID) was determined at Z71, Z79, and Z87 (hard dough) for leaves and grains at final harvest. For leaf sampling, the penultimate leaves of five plants per plot were collected for RLWC and CID measurements at a given stage.

For RLWC determination, the five leaves per plot were cut to a length of approximately 8 cm. The leaves were stored in a closable plastic tube to avoid water loss through transpiration, and then their fresh weight (FW) was immediately determined. Subsequently, the plastic tubes were filled with 1 cm of distilled water, allowing the leaves to reach full turgor. After weighing the turgid weight (TW), the leaves were dried for 24 hours, and the dry weight (DW) was determined. According to Mullan and Pietragalla (2012), the RLWC was then calculated as RLWC = ((FW-DW)/(TW-DW)) × 100. The dried leaf samples were stored for the CID analysis thereafter.

For CID determination for leaves and grains, dried leaf samples from RLWC determination at Z71 and Z79, and from the third leaf sampling at Z87, and grains from final harvest were analyzed for ^13^C discrimination using the mass spectrometer. Consequently, the deviation between the carbon isotope compositions of the samples and the isotopic composition of the PDB standard was measured (Farquhar et al., 1989):


$$\delta [‰]=\frac{R_{p}-R_{s}}{R_{s}}$$

where *R_p_* is the isotopic abundance in the plant and *R_s_* is the abundance ratio ^13^C/^12^C of the standard, for which a fossil from the Pee Dee Formation (Pee Dee Belemnite, PDB) was used (Farquhar et al., 1989).

The carbon isotope discrimination of the plant (*CID*) was then calculated as follows:


$$CID[‰]=\frac{\delta_{a}-\delta_{p}}{1+\delta_{p}}\times 1000,$$

where *δ_a_* is the isotopic composition of the atmosphere (approximately -8‰) and *δ_p_* is the isotopic composition of the plant sample (Farquhar et al., 1989).

### Thermal measurements

The plants’ canopy temperature (CT) was measured with the handheld thermal camera Fluke Ti400 (Fluke Deutschland GmbH, Glottertal, Germany) with a 320 x 240 pixel resolution from the beginning of May until harvest. The measurements were taken weekly in 2017 and 1-2 times per week in 2018 and 2019. As clouds easily bias the plant surface temperature, the thermal pictures were taken only on days without any cloud cover, during the time of day with highest solar radiation (ca 11 a.m. to 1 p.m.). Because of different weather conditions among the three years, there were total 8 x measurements in 2017 and 12 times for both 2018 and 2019, respectively. The camera was held ca. 120 cm above the ground in a nadir position. For analysis, soil and plant pixels were separated using LabView Fluke software (National Instrument v.12.0f3), and only the temperature of the plants was calculated as the mean value for the plot (Rischbeck et al., 2017).

### Calculation of heritability

Heritability was calculated according to Holland et al. (2003). All factors were considered random. Heritability was calculated as follows:


$$h^{2}=\frac{V_{g}}{\left(V_{g}+\frac{V_{r}}{r}\right)}$$

where *V_g_* is the genotypic variance component, *V_r_* is the residual variance component, and *r* is the number of replicates per environment.

### Calculation of rank sums

Rank sums (**Supplementary Material**) were calculated for the grain yield, RLWC at Z79, and CID according to Gad (2010). For each year separately, the data was listed in order using mean values per variety. Each variety received a rank in each year, which were then summed up to calculate the rank sum across all years and irrigation treatments. The rank sums were calculated without the varieties *Slava, Ujinoc, Akteur, Apertus, JB Asano, Kometus, Rumor* and *Tobak d*ue to severe infestation with Tilletia caries in 2019.

### Meteorological data

Meteorological data of Bălți (Moldova) and Freising (Germany) used to compare the different environments was provided by the Agri4cast Resources Portal (Version 3.1) of the Joint Research Centre, European Commission. The grid numbers 94162 (47°49’0.80”N, 27°45’53.71”E, Bălți) and 90114 (48°20’24.50”N, 11°34’15.35”E, Freising) were closest to the respective experimental sites and therefore served for the comparison of the growing conditions at both sites.

### Statistical analysis

Statistical calculations were performed with R studio (version 1.2.5019, RStudio Inc., Boston, MA, USA). Residuals were tested for normal distribution with the Kolmogorov-Smirnov test. For differences between the groups of origin or irrigation treatments, analyses of variance (ANOVA) were calculated (p = 0.05). Groups and means were identified using Tukey’s HSD test. The Pearson correlation coefficient (r) was calculated with significance levels at *p* = 0.05 and *p* = 0.01.

## Results

### Meteorological data

**Figure 2:**
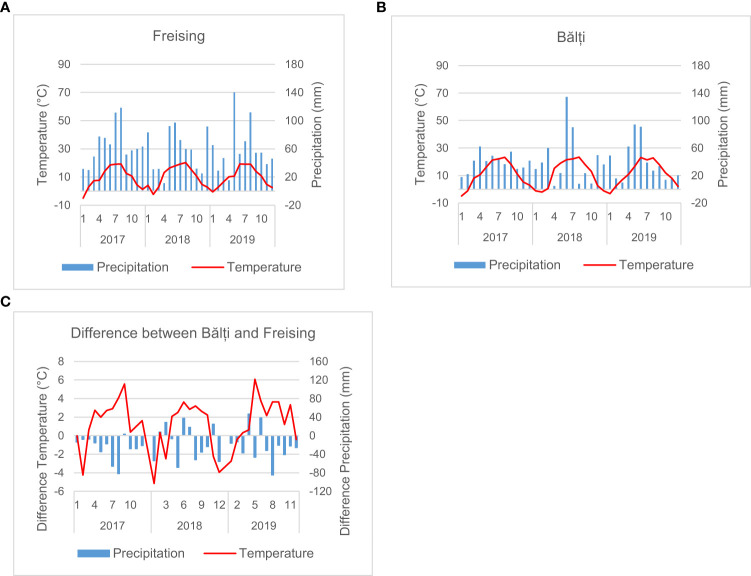
Meteorological conditions in Freising **(A)** and Bălți **(B)**, and the difference between the two locations in the years 2017-2019 **(C)**.

Averaged across the entire growing season, the temperature in Bălți was higher than that in Freising by more than 1.6°C in 2017 and 2019, while the sum of precipitation was at least 254 mm lower (cf. **Figure 2** and **Table 2**). The largest differences were between January 2018 (-5.15°C) and May 2019 (+6.07°C). However, in 2018, the average temperature was only 0.46°C higher than that in South Germany, and precipitation decreased by approximately 180 mm. During the 2017/2018 growing season, the conditions in South Germany and Bălți were more similar than those in other years. During the main growth period between April and July, the temperature difference between Bălți and South Germany was 2.58–3.15°C. The difference in precipitation varied between 134-mm lower precipitation in 2017 and slightly higher precipitation in 2019. Thus, the rainfed conditions in Bălți in this study were considered representing drought/heat stress compared to those in southern Germany.

**Table 2:** Differences of average temperature (T_mean_, °C), sum of precipitation (Ps_um_, mm) and solar radiation (Wh m^-2^) between Balti and Freising within the individual years and within individual growing seasons from April to July.

	2017	2018	2019
T_mean_	1.61	0.46	1.77
P_sum_	-319	-179	-254
Solar radiation	70.3	22.0	72.4
T_mean_ April-July	2.58	2.76	3.15
P_sum_ April-July	-133	-20	6
Solar radiation April-July	35.5	5.4	19.9

In all three years the annual solar radiation was higher in Bălți than in Freising. The difference was smallest in 2018

The annual solar radiation was higher in Bălți than in Freising in all three years, whereas the difference in 2018 was substantially smaller than in 2017 and 2019. Considering only the time from April to July, the values were similar in 2018 but higher in Bălți than in Freising in 2017 and 2019.

### Grain yield

**Figure 3:**
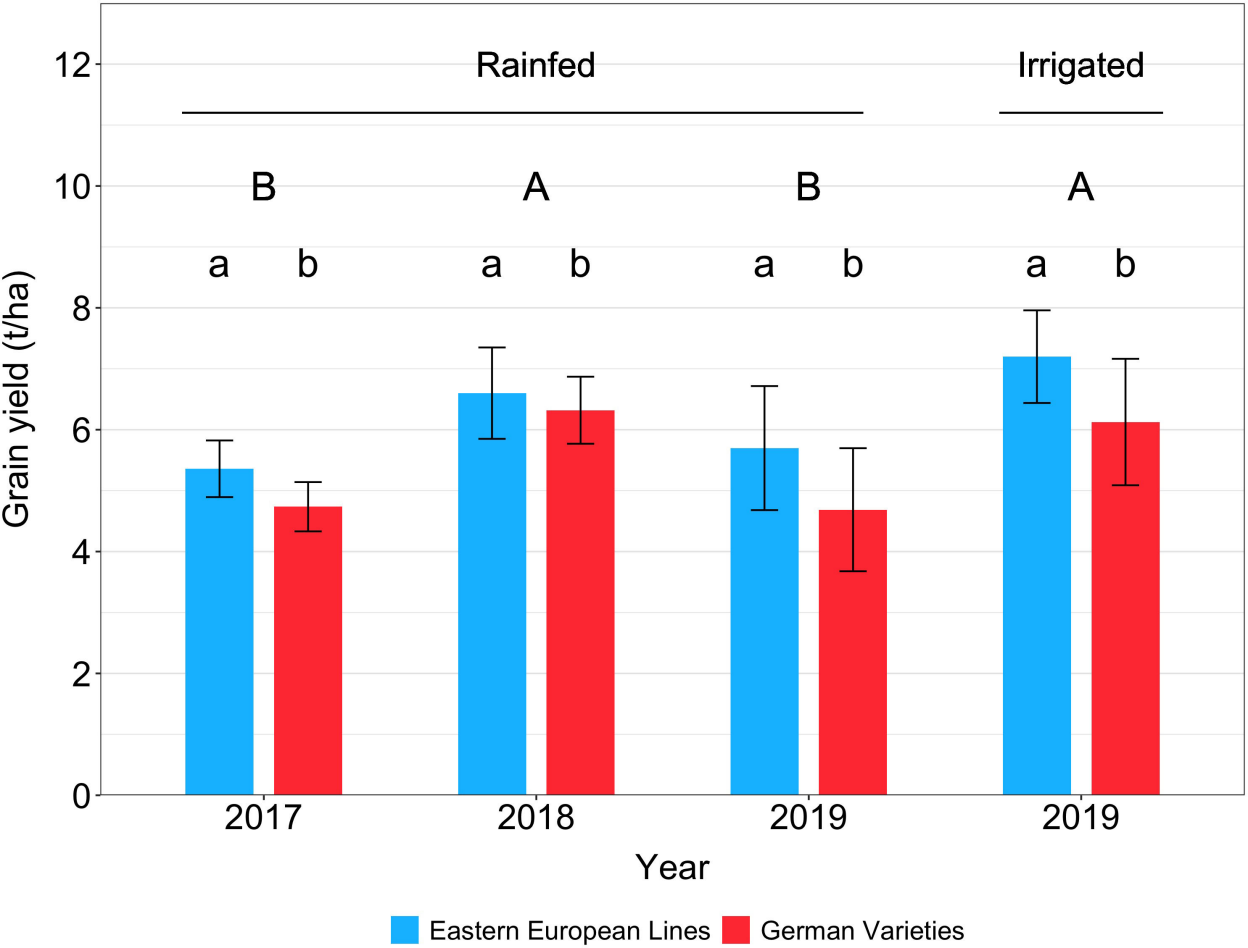
Wheat grain yield of Eastern European lines and German varieties under rainfed conditions in 2017, 2018, and 2019 and under irrigated conditions in 2019. The vertical bars indicate the standard deviation of the mean. Capital letters indicate significant differences between the years, small letters indicate significant differences between origins within one year.

Under rainfed conditions, the yield was 5.4-6.6 t ha^-1^ for Eastern European lines and 4.7-6.3 t ha^-1^ for German varieties (**Figure 3**, **Table 3**). In contrast, the yields measured in the irrigated treatment were 7.2 t ha^-1^ for Eastern European lines, 6.1 t ha^-1^ for German varieties; these yields were significantly higher than those measured under rainfed conditions in 2017 and 2019. The grain yields of the German lines were lower than that of the Eastern European lines, with significant differences (*p* = 0.05) in most cases (**Supplementary Material**, **Figure S1**, **Table S 1**). The yield of the German hybrids was comparable to that of the German lines in 2017 and the Eastern European lines irrigated in 2019. The grain yield in the rainfed treatments was comparable in 2017 and 2019. The same applies to the rainfed treatment in 2018 and the irrigated treatment in 2019. Depending on the origin, no significant difference between any of the variety groups was observed in 2018.

**Table 3:** Analyses of variance and Tukey-HSD test of grain yield, dependent on the origin of varieties.

2017									
Anova							Tukeys HSD		
	Df	Sum Sq	Mean Sq	F value	Pr(>F)			yield_ha	groups
Origin	1	11.63	11.63	61.25	2.40E-12	***	Eastern European	5.36	a
Residuals	118	22.40	0.19				German	4.74	b
Signif. codes: 0 ‘***’ 0.001 ‘**’ 0.01 ‘*’ 0.05 ‘.’ 0.1 ‘ ‘ 1									
2018									
Anova							Tukeys HSD		
	Df	Sum Sq	Mean Sq	F value	Pr(>F)			yield_ha	groups
Origin	1	2.30	2.30	5.27	0.02354	*	Eastern European	6.60	a
Residuals	115	50.15	0.44				German	6.32	b
Signif. codes: 0 ‘***’ 0.001 ‘**’ 0.01 ‘*’ 0.05 ‘.’ 0.1 ‘ ‘ 1									
2019 Rainfed									
Anova							Tukeys HSD		
	Df	Sum Sq	Mean Sq	F value	Pr(>F)			yield_ha	groups
Origin	1	24.16	24.17	23.48	4.96E-06	***	Eastern European	5.70	a
Residuals	94	96.73	1.03				German	4.69	b
Signif. codes: 0 ‘***’ 0.001 ‘**’ 0.01 ‘*’ 0.05 ‘.’ 0.1 ‘ ‘ 1									
2019 irrigated									
Anova							Tukeys HSD		
	Df	Sum Sq	Mean Sq	F value	Pr(>F)			yield_ha	groups
Origin	1	27.18	27.18	34.19	7.20E-08	***	Eastern European	7.20	a
Residuals	94	74.74	0.80				German	6.13	b

Signif. codes: 0 ‘***’ 0.001 ‘**’ 0.01 ‘*’ 0.05 ‘.’ 0.1 ‘ ‘ 1.

### Relative leaf water content (RLWC)

**Figure 4:**
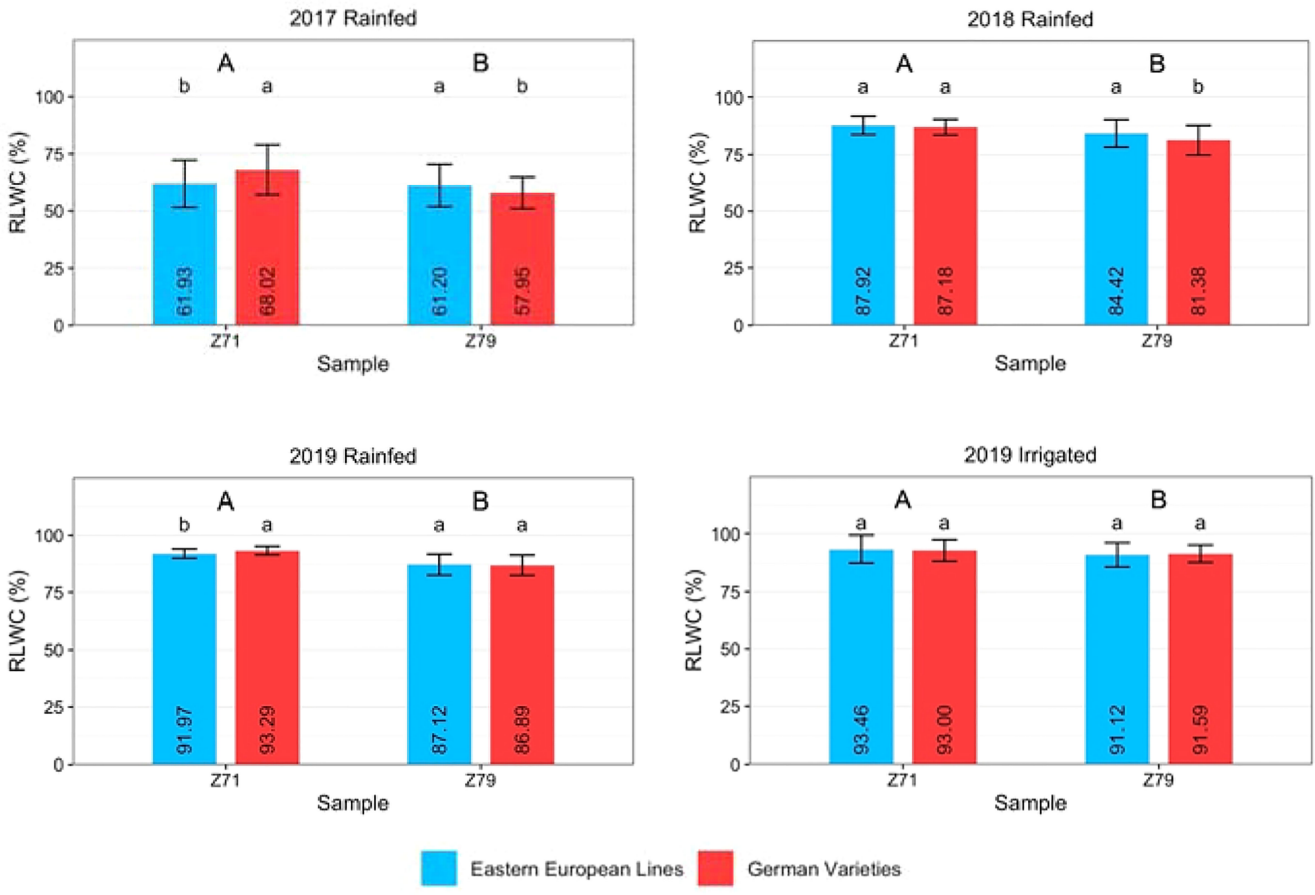
Relative leaf water content (RLWC) of Eastern European lines and German varieties measured at growth stages Z71 and Z79 under rainfed conditions in 2017, 2018, and 2019 and under irrigated conditions in 2019. The vertical bars indicate the standard deviation of the mean. Capital letters indicate significant differences between the years, small letters indicate significant differences between origins within one year.

The RLWC was determined at Z71 and Z79. **Figure 4** and **Table S3** show that RLWC was significantly lower at Z79 than at Z71, regardless of year or treatment. Under rainfed conditions, the RLWC of Eastern European lines was significantly lower at anthesis in 2017 and 2019 than that of German varieties. There was no difference in the RLWC of German hybrids compared to German or Eastern European lines in 2017 or to German lines in 2019 (**Figure S2**). The RLWC of German lines and hybrids exhibited lower values at Z79 than those of Eastern European lines in all rainfed treatments. The only exception was found for the RLWC of German hybrids in 2019, which was slightly higher than that of Eastern European lines.

The correlations between all varieties’ RLWC and grain yield values were low, negative, and nonsignificant, ranging from -0.01 to -0.28 (**Table 4**). Similar trends were found for Eastern European lines. For German wheat, negative and positive correlations, especially for German hybrids, were found across the years.

**Table 4:** Correlation between relative leaf water content (RLWC) measured at Z71 and Z79 and wheat grain yield (Eastern European lines, German hybrids, German lines, and all varieties) under rainfed and irrigated field conditions in Moldova in 2017, 2018 and 2019.

Wheat varieties	Rainfed												Irrigated			
	2017				2018				2019				2019			
	Z71		Z79		Z71		Z79		Z71		Z79		Z71		Z79	
Eastern European lines	-0.15	ns	-0.37	ns	0.03	ns	-0.21	ns	0.14	ns	0.05	ns	-0.17	ns	-0.38	ns
German hybrids	-0.92	ns	-0.97	*	0.12	ns	0.93	ns	0.62	ns	0.82	ns	0.98	*	0.82	ns
German lines	0.58	ns	0.00	ns	-0.01	ns	0.15	ns	-0.68	*	-0.42	ns	-0.26	ns	-0.31	ns
All varieties	-0.16	ns	-0.20	ns	-0.04	ns	-0.20	ns	-0.12	ns	-0.01	ns	-0.05	ns	-0.28	ns

ns, not significant; * = p< 0.05.

### Canopy temperature (CT)

Since CT is very sensitive to prevailing environmental conditions and changes quickly, integrated CT values are less affected by day-to-day variations. **Table 5** shows the accumulative CT by summing all CT measurements from anthesis until final harvest. In 2017 and 2018, there was no significant difference in accumulative CT under rainfed conditions among the three varieties groups. In 2019, however, German hybrids showed significantly lower cumulative CT than Eastern European wheat lines under rainfed and irrigated conditions.

**Table 5:** Accumulative canopy temperature (CT) of Eastern European lines, German hybrids, and German lines measured with a handheld camera from anthesis until harvest under rainfed conditions in 2017, 2018, and 2019 and under irrigated conditions in 2019.

	Rainfed												Irrigated			
	2017				2018				2019				2019			
Eastern European lines	214.46	±	4.29	a	289.63	±	3.89	a	339.00	±	5.39	a	323.45	±	3.81	a
German hybrids	216.95	±	4.08	a	288.32	±	4.33	a	328.46	±	11.98	b	315.52	±	8.55	b
German lines	214.95	±	5.24	a	289.63	±	5.26	a	336.50	±	6.82	ab	318.88	±	4.00	ab

The vertical bars indicate the standard deviation of the mean. Different letters show significance between origins within the individual years and treatments.

The correlations between the accumulated CT and the grain yield (**Table S2**) ranged from -0.69 to 0.27 and were not significant. German wheat varieties showed a higher correlation in 2018 and 2019 than Eastern European lines.

**Table:** 

Wheat varieties	Rainfed						Irrigated	
	2017		2018		2019		2019	
Eastern European lines	-0.39	ns	0.27	ns	-0.26	ns	-0.06	ns
German hybrids	0.23	ns	-0.48	ns	-0.69	ns	-0.60	ns
German lines	0.08	ns	-0.51	ns	-0.58	ns	-0.20	ns
All varieties	-0.21	ns	-0.04	ns	-0.58	ns	-0.20	ns

### Carbon isotope discrimination (CID)

**Figure 5:**
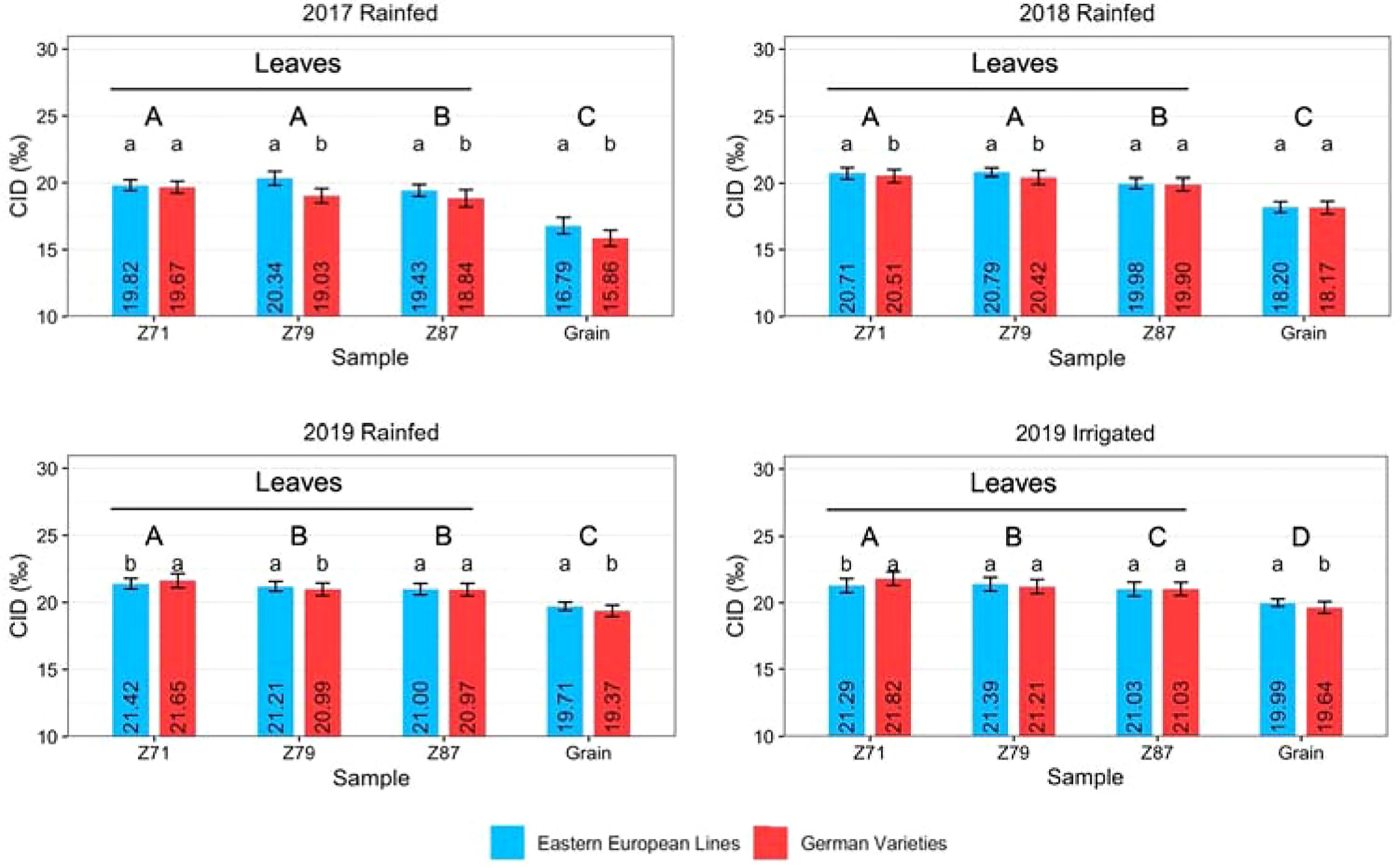
Carbon isotope discrimination (CID) of wheat leaves at Z71, Z79, and Z87 and grains of Eastern European lines and German varieties under rainfed conditions in 2017, 2018, and 2019 and under irrigated conditions in 2019. The vertical bars indicate the standard deviation of the mean. Capital letters indicate significant differences between the years, small letters indicate significant differences between origins within one year.

The rate of CID showed a similar pattern across all trials (**Figure 5**). The difference between leaf and grain samples was significant in all years. At most sampling times, the difference between Eastern European and German varieties was significant. Only in 2019, both in the rainfed and irrigated trials, at Z71 the Eastern European lines showed a significantly lower CID than the German varieties. German lines showed a lower CID than German hybrids in most cases (**Figure S3**). The difference in CID between Z71 and grains at harvest was the smallest for Eastern European lines in most cases (2017: 3.03 ‰, 2019 rainfed: 1.71 ‰, and 2019 irrigated: 1.3 ‰). Only in 2018, German varieties 2.34 ‰) showed a smaller difference between the first and the last sampling than the Eastern European lines (2.51 ‰). The difference between 2017 and 2018 was larger than that between the rainfed and irrigated treatments in 2019 for Eastern and German varieties.

**Table 6** shows the correlation between the rate of CID and grain yield. When comparing the years and single groups of varieties, the most significant correlations were found in 2017; however, none were obtained for Eastern European lines and leaves at Z71. In 2018, only the CID of grains of hybrid varieties was significantly correlated with yield. Under rainfed and irrigated conditions in 2019, significant correlations were found for Z87 (eastern European lines, rainfed) and grains regardless of the variety groups. When comparing the three groups of varieties across three years, the best correlations were obtained for German hybrids, followed by German lines. When the data of all varieties were used to calculate the correlation, more significant differences were found; however, again, in 2018, the lowest number of significant correlations was observed. Higher correlations were obtained at later growth stages of leaves and with grains. Only in the irrigated plots in 2019 was a significant correlation with grain yield found at Z71.

**Table 6:** Correlation between Carbon Isotope Discrimination (CID) in leaves measured at Z71, Z79, and Z87 and in grains and wheat grain yield of wheat variety groups (all varieties, Eastern European lines, German hybrids, and German lines) under rainfed and irrigated field conditions in Moldova in 2017, 2018 and 2019.

Wheat varieties	Rainfed																								Irrigated							
	2017								2018								2019								2019							
	Leaves						Grain		Leaves						Grain		Leaves						Grain		Leaves						Grain	
	Z71		Z79		Z87-89				Z71		Z79		Z87-89				Z71		Z79		Z87-89				Z71		Z79		Z87-89		
**Eastern European lines**	-0.07	ns	0.14	ns	-0.02	ns	0.06	ns	0.06	ns	0.07	ns	0.05	ns	0.06	ns	0.06	ns	0.21	ns	0.33	*	0.01	ns	-0.05	ns	-0.10	ns	-0.05	ns	0.28	*
**German hybrids**	-0.46	ns	0.52	ns	0.70	*	0.59	*	0.10	ns	0.30	ns	0.10	ns	0.68	**	-0.15	ns	0.16	ns	0.38	ns	0.84	**	0.06	ns	-0.17	ns	0.34	ns	0.82	**
**German lines**	0.03	ns	0.45	**	0.63	**	0.20	ns	0.10	ns	0.18	ns	-0.03	ns	0.10	ns	-0.16	ns	0.01	ns	0.08	ns	0.30	*	0.05	ns	-0.08	ns	0.26	ns	0.32	*
**All varieties**	0.07	ns	0.61	**	0.52	**	0.46	**	0.13	ns	0.22	*	0.04	ns	0.12	ns	-0.05	ns	0.30	**	0.30	**	0.46	**	-0.18	*	0.17	ns	0.22	*	0.57	**

ns, not significant; * = p< 0.05; ** = p< 0.01.

### Heritability of grain yield, RLWC, CT, and CID

Grain yield generally showed high heritability (h^2^ = 0.64-0.99, **Table 7**). The heritability of the German hybrids was higher than that of the other variety groups. Among the three physiological traits, the heritability of RLWC was lower than the heritability of CID or CT, regardless of the year. The heritability of accumulative CT for German varieties was higher than that for Eastern European lines in 2017 and 2018. In rainfed and irrigated treatments in 2019, German hybrids showed a high heritability of CT, whereas German and Eastern European lines only showed low to moderate heritability (rainfed: h^2^= 0.27-0.30, irrigated: h^2^= 0.66).

**Table 7:** Heritability of wheat grain yield, relative water content (RLWC), canopy temperature (CT), and carbon isotope discrimination (CID) in leaves (CID_L) and grains (CID_G) of wheat groups (all varieties, Eastern European lines, German hybrids, and German lines) under rainfed and irrigated field conditions in Moldova in 2017, 2018 and 2019.

Wheat varieties	Traits	Rainfed			Irrigated
		2017	2018	2019	2019
Eastern European lines	Yield	0.87	0.85	0.98	0.94
	RLWC	0.63	0.48	0.67	0.73
	CT	0.85	0.53	0.3	0.66
	CID_L	0.46	0.8	0.8	0.79
	CID_G	0.37	0.64	0.39	0.65
German hybrids	Yield	0.91	0.95	0.99	0.98
	RLWC	0.59	0.44	0	0.09
	CT	0.97	0.84	0.9	0.98
	CID_L	0.96	0.91	0	0.94
	CID_G	0.9	0.95	0.86	0.95
German lines	Yield	0.64	0.94	0.94	0.94
	RLWC	0	0.54	0.56	0.63
	CT	0.94	0.94	0.27	0.66
	CID_L	0.79	0.91	0.84	0.9
	CID_G	0.62	0.88	0.71	0.88
All varieties	Yield	0.88	0.89	0.98	0.97
	RLWC	0.43	0.53	0.59	0.61
	CT	0.83	0.8	0.56	0.84
	CID_L	0.89	0.89	0.82	0.87
	CID_G	0.76	0.82	0.76	0.88

In most cases, the CID of leaves and grain was higher for German varieties than for Eastern European varieties. The CID showed higher heritability in leaves than in grains and higher heritability for German lines and hybrids than Eastern European lines.

## Discussion

Falconer (1989) recommended that high heritability and strong correlations between indirect traits and grain yield are desirable to evaluate the reliability of indirect traits for plant breeding. Among the three physiological traits of the RLWC, CT, and CID that characterize the plant water status under drought/heat conditions, the heritability of RLWC showed the lowest values regardless of year and variety group (**Table 7**). The RLWC across all varieties did not correlate with grain yield within individual years in this study (**Table 5**), suggesting that the RLWC did not seem to be a useful trait in distinguishing origins or varieties. Furthermore, although heritability demonstrated high values (**Table 7**), there were no significant correlations between CT and grains (**Table S5**).

In contrast, the heritability of CID of leaves (h^2^= 0.69-0.74) and grain (h^2^= 0.64-0.65) (Table 7) was high, which was even comparable to h^2^ of grain yield in most cases. High heritability for grain CID has been reported by Merah et al. (2001) and Becker and Schmidhalter (2017). The results of this study indicate that CID is an important breeding target for improving plant water use efficiency and yield under rainfed conditions. Furthermore, significant correlations between grain yield and CID were found in most cases (**Table 6**). Together with the heritability of CID in both leaves and grains, this study suggests that CID, as an indirect physiological trait, performs much better than CT and RLWC. Several studies on wheat have shown a close correlation between CID and final grain yield under drought conditions. Correlations between CID and grain yield are normally high, either negative or positive, according to the plant tissue sampled and environmental conditions tested (Condon and Richards, 1992; Voltas et al., 1999; Araus et al., 2003). Previous studies have found a positive correlation between CID and grain yield while testing a similar panel of German wheat cultivars under rain-out shelter conditions in Germany (Becker and Schmidhalter, 2017). In this study, German hybrids and lines showed significant correlations between CID and grain yield, particularly for CID in grains, whereas the Eastern European varieties only showed low correlations (**Table 6**). Severe stress led to higher variability in the stress response, leading to stronger correlations due to the scattering of the data. With higher precipitation (2017/2018) or irrigation (2018/2019), the differences between the varieties decreased, and possible correlations were more difficult to detect. The same effect could be observed for Eastern European varieties due to a higher degree of adaptation, thereby decreasing the correlation. Within the set of German varieties, it was possible to see a correlation between the rate of CID and grain yield. Considering the CID, this was also the case in the growing season of 2018/2019, but the stress level in this year was lower than in the first and second years of study.

  CID values may help to determine the degree of drought stress (Condon et al., 1990) because plants under drought stress close their stomata to reduce transpirational water loss that limits CO_2_ uptake (Medrano, 2002; Farooq et al., 2012), leading to an increasing ^13^C:^12^C ratio and a reduction in carbon isotope discrimination (Farquhar et al., 1989). Therefore, a higher rate of CID indicates less physiological stress, less damage to plant tissue, and more assimilation, which together lead to increased grain yields (Farquhar and Richards, 1984; Condon et al., 2004). In this study, CID values depended on the varieties´ origin and stress conditions. In 2017, the CID values of leaves and grains were lowest compared to the other years, indicating that the plants were under the highest level of stress (**Figure 5**). In contrast, the difference between the rainfed treatment in 2018 and both the rainfed and irrigated treatments in 2019 was much smaller. A high level of stress caused lower CID in leaves and grains. These results are consistent with the recorded meteorological data: the 2016/2017 season had the lowest total precipitation (**Table 2**) and the lowest ratio between precipitation and mean temperature (Kunz, 2021).

Furthermore, in 2017 and 2018, the Eastern European lines showed a higher rate of CID than the German varieties for most samples. In 2019, the CID of German hybrids was comparable to or higher than that of the Eastern European lines. The meteorological data indicate that the Eastern European lines could better withstand a higher stress level in 2017 and 2018 than the German varieties. This could imply either a closure of stomata to reduce water loss while tolerating increased tissue temperature or the ability to keep stomata open and tolerate a lower water potential. Eastern European varieties had a slightly higher CT than German varieties (**Table 5**), so they seem to close their stomata. However, both strategies are likely used to a certain level. In 2019, the stress level was lower than in the previous years and more water from precipitation could be stored in the soil, resulting in higher CID values. Due to the relatively high amount of precipitation during the growing season, the difference between rainfed and irrigated plots was not as large. The largest difference between the three years was reflected by increased CID values of German hybrid varieties in 2019 (**Figure 5**). Hybrid varieties can often tolerate abiotic stress better than line varieties (Schittenhelm et al., 2019), which could be one of the reasons for a higher CID in this study.

Throughout the study, CID decreased with growth stages from the first sampling to the grain harvest (**Figure 5**). In all years, the CID decreased from the first leaf sampling to the final harvest. It is generally known that the carbohydrates stored in the upper leaf levels contribute to grain filling to a large extent (Lupton, 1966). However, the difference between the CID of leaves at Z87 and of grains was significant regardless of year. This might arise because the carbohydrates exported to grains have a different isotopic composition than those remaining in the leaves or because the grains accumulate carbon not only from the leaves but also from other source organs, such as stems (Merah et al., 2001). The largest difference between the three years was the increased CID of German hybrid varieties in 2019 (**Figure S 3**). A high degree of CID indicates the ability of the plants to keep the stomata open and to have a higher stomatal conductance (Farooq et al., 2012). Although the differences were not always significant, it can be concluded that the hybrids maintained higher photosynthetic activity in 2019 than the two groups of line varieties. These assumptions are supported by the high plant dry weight at anthesis and harvest, plant height, and grain size distribution (Kunz, 2021). The grain yield of hybrids was comparable to that of Eastern European lines and significantly higher than that of German lines in irrigated plots (**Figure S1**).

The CT related to water use and crop yield formation is considered an important trait to select for adapted genotypes. Further advances in regular nondestructive assessments of CT during the breeding process hold great promise for the indirect selection of varieties in a high-throughput manner (Rischbeck et al., 2016). Although the correlations between CT and grain yield were low and insignificant in most cases (**Table S5**), CT generally showed a level of heritability comparable to CID, indicating that CT could be used as a secondary trait in selection processes as well. This supports the findings of Amani et al. (1996); Becker and Schmidhalter (2017), and Deery et al. (2016). However, some positive correlations between CT and grain yield for the Eastern European lines contradict the aforementioned physiological principles. In this case, it must be taken into account that the Eastern European varieties are much better adapted to the climate in northern Moldova than the German varieties. To find a possible relation between CT and grain yield, the varieties should be compared within one origin group rather than across the origins. The varieties used for the field experiment in Moldova had different genetic backgrounds, therefore rank sums of grain yield, RLWC and CID were calculated highlighting the performance of individual varieties (**Tables S2**, **S4**, **S7**).

The low and nonsignificant correlations between CT and grain yield may have resulted from CT being sensitive to prevailing environmental conditions. CT measurements are influenced by daily temperature and alter under changing weather conditions, whereas CID values integrates over time. Although only pixels representing the plant surface were used when calculating the average temperature, it is likely that the dark soil heated up, radiated heat, and increased the temperature of the plant surface, thus supplementing the direct solar radiation received from above (Deery et al., 2016; Rischbeck et al., 2017). Another difficulty was that differentiating colors between plant surfaces and soil pixels became increasingly challenging with senescence, and this could easily lead to biased results. As senescent plants are not able to control their surface temperature any longer, progressing senescence bears the risk of a bias in CT measurements.

CT is sensitive to cloud cover and to the sunshine intensity. Although thermal pictures were taken only on cloudless days, the radiation intensity might have varied among the days, and the measurement duration was still fairly long (ca. 40 min per 120 plots), so the conditions were not equal for all images. These difficulties need to be taken into consideration when judging the results. Integrated values are less affected by day-to-day variations and, therefore, might have more explanatory power and show stronger correlations.

When measured destructively, RLWC is also very sensitive to prevailing environmental conditions and can change quickly. In addition, RLWC sampling always runs the risk of being falsified by the water losses that can occur during the handling or weighing processes. These losses may lead to low heritability (**Table 7**) and correlations (**Table 4**) and inconsistent results among the years and variety groups of study, especially positive correlations between grain yield and RLWC. Thus, this study recommends that RLWC field measurements will be considered an unreliable indirect trait.

## Conclusions

The traits of RLWC, CT, and CID for plant drought/heat tolerance can serve as indirect selection criteria to improve grain yields. This study evaluated the performance of CID, CT, and RLWC to phenotype drought/heat resistance of different origins of winter wheat at similar growth stages under natural stress conditions. It can be concluded that CID can be used as a phenotyping approach for drought/heat adaptation under field conditions. In contrast, RLWC did not seem to be a useful trait for distinguishing origins or varieties. To phenotype RLWC traits under field conditions, destructive RLWC sampling always faces the risk of being falsified by unavoidable water loss during the handling and weighing processes, especially to sample many plots. Although the trait of CT has been considered a promising approach for high-throughput phenotyping of plant drought/heat tolerance, the current study found low and nonsignificant correlations between CT and grain yield, although the heritability of CT demonstrated high values. This finding was possibly due to the high sensitivity of CT to the prevailing environmental conditions and seems particularly challenging in dark Chernozem soils since they heat up, radiate heat, and may increase the temperature of plant surfaces requiring further research.

## Data availability statement

The raw data supporting the conclusions of this article will be made available by the authors, without undue reservation.

## Author contributions

US and YH conceived the original research. KK designed the experiments. KK performed the experiments, analyzed the data, and wrote the draft manuscript. US supervised the experiments. YH and US revised the manuscript. All authors contributed to the article and approved the submitted version.

## Funding

This research was supported by the German Research Foundation (DFG) funds for project No. SCHM 1456/8-1.

## Conflict of interest

The authors declare that the research was conducted in the absence of any commercial or financial relationships that could be construed as a potential conflict of interest.

## Publisher’s note

All claims expressed in this article are solely those of the authors and do not necessarily represent those of their affiliated organizations, or those of the publisher, the editors and the reviewers. Any product that may be evaluated in this article, or claim that may be made by its manufacturer, is not guaranteed or endorsed by the publisher.
