# Supplementary material for: Carbon isotope discrimination as a key physiological trait to phenotype drought/heat resistance of future climate-resilient German winter wheat compared with relative leaf water content and canopy temperature
Source: Front Plant Sci. 2022 Nov 30;13:1043458. doi: 10.3389/fpls.2022.1043458 (PMC9794500; doi:10.3389/fpls.2022.1043458)
Supplement: Supplementary file 1 [file DataSheet_1.docx]

# Supplementary Material


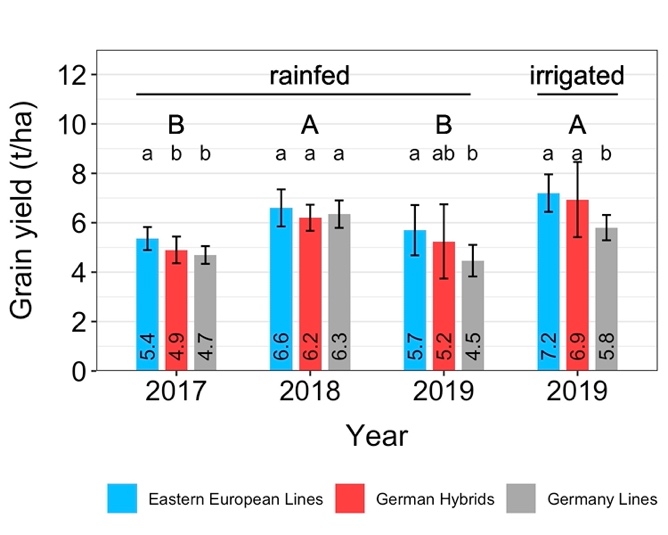


Figure S 1 Wheat grain yield of Eastern European lines and German varieties under rainfed conditions in 2017, 2018, and 2019 and under irrigated conditions in 2019. The vertical bars indicate the standard deviation of the mean. Capital letters indicate significant differences between the years, small letters indicate significant differences between origins within one year.

Table S 1 Analyses of variance and Tukey-HSD test of grain yield, dependent on the origin and breed of varieties.

| 2017 |  |  |  |  |  |  |  |  |  |  |
| --- | --- | --- | --- | --- | --- | --- | --- | --- | --- | --- |
| Anova | | | | | | |  | Tukeys HSD | | |
|  | Df | Sum Sq | Mean Sq | F value | Pr(>F) |  |  |  | yield_ha | groups |
| origin_breed | 2 | 12.028 | 6.0139 | 31.987 | 8.29E-12 | *** |  | Eastern European Line | 5.35861 | a |
| Residuals | 117 | 21.997 | 0.188 |  |  |  |  | German Hybrid | 4.8998 | b |
|  |  |  |  |  |  |  |  | German Line | 4.695167 | b |
|  |  |  |  |  |  |  |  |  |  |  |
|  |  |  |  |  |  |  |  |  |  |  |
| 2018 |  |  |  |  |  |  |  |  |  |  |
| Anova | | | | | | |  | Tukeys HSD | | |
|  | Df | Sum Sq | Mean Sq | F value | Pr(>F) |  |  |  | yield_ha | groups |
| origin_breed | 2 | 2.489 | 1.2446 | 2.8402 | 0.06256 | . |  | Eastern European Line | 6.599673 | a |
| Residuals | 114 | 49.955 | 0.4382 |  |  |  |  | German Hybrid | 6.34775 | a |
|  |  |  |  |  |  |  |  | German Line | 6.200609 | a |
|  |  |  |  |  |  |  |  |  |  |  |
|  |  |  |  |  |  |  |  |  |  |  |
|  | |  |  |  |  |  |  |  |  |  |
| Anova | | | | | | |  | Tukeys HSD | | |
|  | Df | Sum Sq | Mean Sq | F value | Pr(>F) |  |  |  | yield_ha | groups |
| origin_breed | 2 | 29.356 | 14.678 | 14.912 | 2.41E-06 | *** |  | Eastern European Line | 5.698034 | a |
| Residuals | 93 | 91.541 | 0.9843 |  |  |  |  | German Hybrid | 5.242515 | ab |
|  |  |  |  |  |  |  |  | German Line | 4.464322 | b |
|  |  |  |  |  |  |  |  |  |  |  |
|  |  |  |  |  |  |  |  |  |  |  |
| 2019 irrigated | |  |  |  |  |  |  |  |  |  |
| Anova | | | | | | |  | Tukeys HSD | | |
|  |  |  |  |  |  |  |  |  | yield_ha | groups |
| origin_breed | 2 | 38.269 | 19.1343 | 27.957 | 3.11E-10 | *** |  | Eastern European Line | 7.198793 | a |
| Residuals | 93 | 63.651 | 0.6844 |  |  |  |  | German Hybrid | 6.938392 | a |
|  |  |  |  |  |  |  |  | German Line | 5.801199 | b |
| Signif. codes: 0 ‘***’ 0.001 ‘**’ 0.01 ‘*’ 0.05 ‘.’ 0.1 ‘ ’ 1 | | | | | | | | | | |

Table S 2 Rank sum of grain yield across all years. Sorted from highest to lowest rank sum within the groups of origin.

| Variety | Origin | Breed | Grain Yield 2017 Rainfed | Rank 2017 Rainfed | Grain Yield 2018 Rainfed | Rank 2018 Rainfed | Grain Yield 2019 Rainfed | Rank 2019 Rainfed | Grain Yield 2019 Irrigated | Rank 2019 Irrigated | Rank sum Grain Yield |
| --- | --- | --- | --- | --- | --- | --- | --- | --- | --- | --- | --- |
| Ursita | East | Line | 5.14 | 16 | 7.39 | 1 | 7.93 | 1 | 7.99 | 5 | 23 |
| Pajura | East | Line | 5.42 | 9 | 7.06 | 7 | 6.32 | 6 | 8.32 | 3 | 25 |
| Zisk | East | Line | 6.01 | 1 | 6.80 | 12 | 6.68 | 5 | 7.75 | 7 | 25 |
| Meleag | East | Line | 5.80 | 3 | 6.56 | 19 | 5.96 | 8 | 7.70 | 8 | 38 |
| Rowina | East | Line | 5.85 | 2 | 6.33 | 21 | 6.14 | 7 | 7.41 | 10 | 40 |
| Zolotocolosa | East | Line | 5.52 | 7 | 7.37 | 2 | 4.83 | 20 | 7.05 | 12 | 41 |
| Amor | East | Line | 5.67 | 5 | 7.03 | 8 | 5.45 | 12 | 6.64 | 16 | 41 |
| Unitar | East | Line | 4.33 | 31 | 7.26 | 3 | 6.82 | 4 | 7.90 | 6 | 44 |
| Semnal | East | Line | 4.60 | 27 | 6.72 | 15 | 7.24 | 3 | 8.38 | 2 | 47 |
| Transitor | East | Line | 5.80 | 4 | 6.76 | 13 | 5.32 | 13 | 6.46 | 19 | 49 |
| Kuialnik | East | Line | 4.93 | 19 | 7.13 | 5 | 5.15 | 17 | 7.41 | 9 | 50 |
| FGmut 293 | East | Line | 5.54 | 6 | 6.58 | 18 | 5.18 | 15 | 6.91 | 14 | 53 |
| Acord | East | Line | 5.31 | 12 | 7.07 | 6 | 4.34 | 27 | 7.33 | 11 | 56 |
| Clasic | East | Line | 5.19 | 15 | 6.35 | 20 | 5.77 | 10 | 6.87 | 15 | 60 |
| Numitor | East | Line | 5.33 | 11 | 6.72 | 14 | 4.39 | 26 | 6.95 | 13 | 64 |
| Talisman | East | Line | 5.39 | 10 | 6.17 | 25 | 4.69 | 21 | 6.50 | 18 | 74 |
| Savant | East | Line | 5.00 | 18 | 6.22 | 24 | 5.69 | 11 | 5.89 | 25 | 78 |
| Zagrava | East | Line | 5.27 | 13 | 5.36 | 31 | 4.66 | 22 | 6.10 | 22 | 88 |
| Hyfi | Ger | Hybrid | 5.44 | 8 | 6.68 | 17 | 5.92 | 9 | 8.39 | 1 | 35 |
| Hybery | Ger | Hybrid | 4.69 | 25 | 6.07 | 28 | 7.25 | 2 | 8.31 | 4 | 59 |
| Mulan | Ger | Line | 4.86 | 21 | 6.84 | 10 | 5.22 | 14 | 6.43 | 20 | 65 |
| Elixer | Ger | Line | 5.09 | 17 | 6.96 | 9 | 5.03 | 18 | 6.00 | 23 | 67 |
| Discus | Ger | Line | 4.50 | 28 | 7.25 | 4 | 4.42 | 25 | 5.92 | 24 | 81 |
| Colonia | Ger | Line | 4.76 | 23 | 6.82 | 11 | 3.61 | 31 | 6.50 | 17 | 82 |
| Genius | Ger | Line | 4.71 | 24 | 6.09 | 26 | 5.15 | 16 | 6.28 | 21 | 87 |
| Kerubino | Ger | Line | 4.85 | 22 | 6.70 | 16 | 4.47 | 24 | 5.14 | 32 | 94 |
| Patras | Ger | Line | 4.89 | 20 | 5.96 | 29 | 4.85 | 19 | 5.64 | 27 | 95 |
| Hystar | Ger | Hybrid | 5.19 | 14 | 4.44 | 32 | 4.08 | 28 | 5.78 | 26 | 100 |
| Impression | Ger | Line | 4.64 | 26 | 6.28 | 22 | 4.50 | 23 | 5.36 | 29 | 100 |
| Manager | Ger | Line | 4.48 | 30 | 6.07 | 27 | 3.83 | 29 | 5.43 | 28 | 114 |
| Anapolis | Ger | Line | 4.48 | 29 | 6.22 | 23 | 3.57 | 32 | 5.30 | 30 | 114 |
| Hybred | Ger | Hybrid | 4.28 | 32 | 5.54 | 30 | 3.72 | 30 | 5.27 | 31 | 123 |
| EE: Eastern European varieties, Ger: German varieties. | | | | | | | | | | | |

| 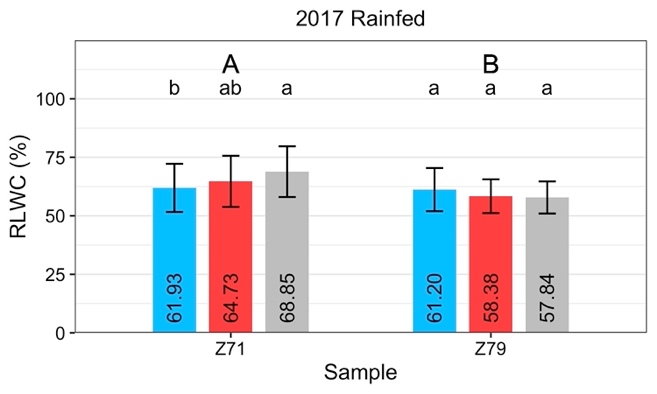 | 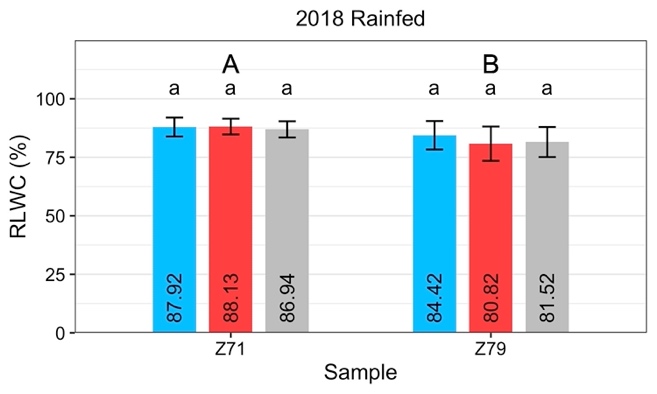 |
| --- | --- |
| 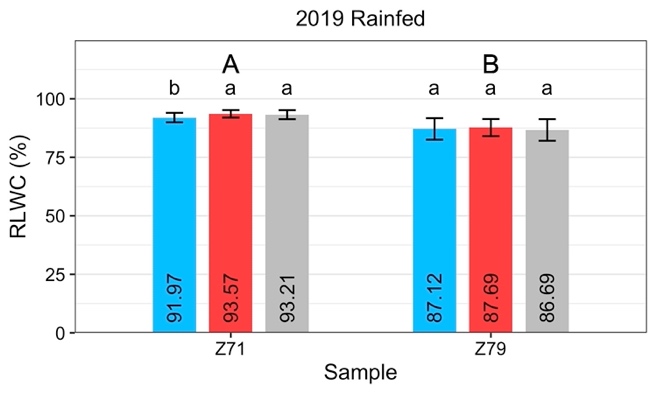 | 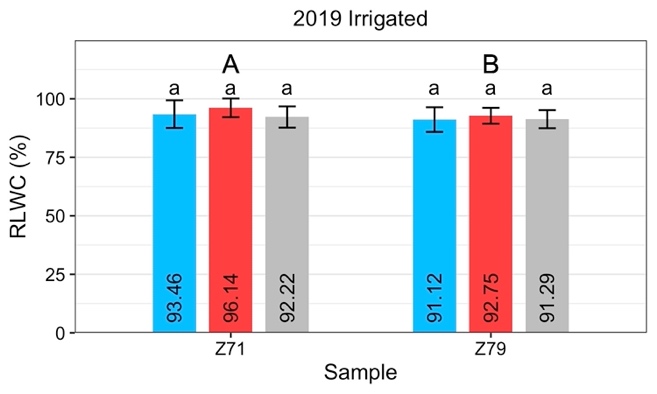 |
| 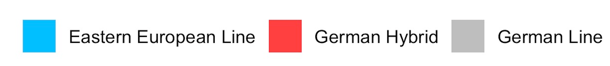 | |

Figure S 2 Relative leaf water content (RLWC) of Eastern European lines, German hybrids and German lines measured at growth stages Z71 and Z79 under rainfed conditions in 2017, 2018, and 2019 and under irrigated conditions in 2019. The vertical bars indicate the standard deviation of the mean. Capital letters indicate significant differences between the years, small letters indicate significant differences between origins within one year.

Table S 3 Analyses of variance and Tukey-HSD test of RLWC, dependent on the origin of varieties.

| 2017 |  |  |  |  |  |  |  |  |  | |  |
| --- | --- | --- | --- | --- | --- | --- | --- | --- | --- | --- | --- |
| Samples |  |  |  |  |  |  |  |  |  | |  |
| Anova | | | | | | |  | Tukeys HSD | | | |
|  | Df | Sum Sq | Mean Sq | F value | Pr(>F) |  |  |  | X2017 | | groups |
| sample | 1 | 1711.5 | 1711.54 | 18.124 | 2.99E-05 | *** |  | Z71 | 64.94879 | | a |
| Residuals | 235 | 22192.2 | 94.44 |  |  |  |  | Z79 | 59.57411 | | b |
|  |  |  |  |  |  |  |  |  |  | |  |
| Z71 |  |  |  |  |  |  |  |  |  | |  |
| Anova | | | | | | |  | Tukeys HSD | | | |
|  | Df | Sum Sq | Mean Sq | F value | Pr(>F) |  |  |  | X2017 | | groups |
| origin | 1 | 1100.3 | 1100.29 | 9.8148 | 0.002188 | ** |  | Germany | 68.0152 | | a |
| Residuals | 117 | 13116.3 | 112.11 |  |  |  |  | Eastern Europe | 61.93349 | | b |
|  |  |  |  |  |  |  |  |  |  | |  |
| Z79 |  |  |  |  |  |  |  |  |  | |  |
| Anova | | | | | | |  | Tukeys HSD | | | |
|  | Df | Sum Sq | Mean Sq | F value | Pr(>F) |  |  |  | X2017 | | groups |
| origin | 1 | 311.1 | 311.106 | 4.7085 | 0.03205 | * |  | Eastern Europe | 61.19784 | | a |
| Residuals | 116 | 7664.5 | 66.073 |  |  |  |  | Germany | 57.95038 | | b |
|  |  |  |  |  |  |  |  |  |  | |  |
|  |  |  |  |  |  |  |  |  |  | |  |
| 2018 |  |  |  |  |  |  |  |  |  | |  |
| Samples |  |  |  |  |  |  |  |  |  | |  |
| Anova | | | | | | |  | Tukeys HSD | | | |
|  | Df | Sum Sq | Mean Sq | F value | Pr(>F) |  |  |  | X2018 | | groups |
| origin | 1 | 1288 | 1288.04 | 45.856 | 9.94E-11 | *** |  | Z71 | 87.54276 | | a |
| Residuals | 237 | 6657 | 28.09 |  |  |  |  | Z79 | 82.89975 | | b |
|  |  |  |  |  |  |  |  |  |  | |  |
| Z71 |  |  |  |  |  |  |  |  |  | |  |
| Anova | | | | | | |  | Tukeys HSD | | | |
|  | Df | Sum Sq | Mean Sq | F value | Pr(>F) |  |  |  | X2018 | | groups |
| origin | 1 | 16.31 | 16.307 | 1.1564 | 0.2844 |  |  | Eastern Europe | 87.91607 | | a |
| Residuals | 117 | 1649.85 | 14.101 |  |  |  |  | Germany | 87.17568 | | a |
|  |  |  |  |  |  |  |  |  |  | |  |
| Z79 |  |  |  |  |  |  |  |  |  | |  |
| Anova | | | | | | |  | Tukeys HSD | | | |
|  | Df | Sum Sq | Mean Sq | F value | Pr(>F) |  |  |  | X2018 | | groups |
| origin | 1 | 277.1 | 277.138 | 6.9377 | 0.009571 | ** |  | Eastern Europe | 84.41945 | | a |
| Residuals | 118 | 4713.7 | 39.947 |  |  |  |  | Germany | 81.38006 | | b |
|  |  |  |  |  |  |  |  |  |  | |  |
|  |  |  |  |  |  |  |  |  |  | |  |
| 2019 Rainfed |  |  |  |  |  |  |  |  |  | |  |
| Samples |  |  |  |  |  |  |  |  |  | |  |
| Anova | | | | | | |  | Tukeys HSD | | | |
|  | Df | Sum Sq | Mean Sq | F value | Pr(>F) |  |  |  | X2019.dry | | groups |
| sample | 1 | 1874.8 | 1874.8 | 154.94 | < 2.20E-16 | *** |  | Z71 | 92.62836 | | a |
| Residuals | 235 | 2843.5 | 12.1 |  |  |  |  | Z79 | 87.00273 | | b |
|  |  |  |  |  |  |  |  |  |  | |  |
| Z71 |  |  |  |  |  |  |  |  |  | |  |
| Anova | | | | | | |  | Tukeys HSD | | | |
|  | Df | Sum Sq | Mean Sq | F value | Pr(>F) |  |  |  | X2019.dry | | groups |
| origin | 1 | 51.78 | 51.782 | 13.639 | 0.000337 | *** |  | Germany | 93.28526 | | a |
| Residuals | 118 | 448 | 3.797 |  |  |  |  | Eastern Europe | 91.97146 | | b |
| Signif. codes: 0 ‘***’ 0.001 ‘**’ 0.01 ‘*’ 0.05 ‘.’ 0.1 ‘ ’ 1 | | | | | | | | | | | |
|  |  |  |  |  |  |  |  |  |  | |  |
| Z79 |  |  |  |  |  |  |  |  |  | |  |
| Anova | | | | | | |  | Tukeys HSD | | | |
|  | Df | Sum Sq | Mean Sq | F value | Pr(>F) |  |  |  | X2019.dry | | groups |
| origin | 1 | 1.58 | 1.5789 | 0.0775 | 0.7812 |  |  | Eastern Europe | 87.12192 | | a |
| Residuals | 115 | 2342.17 | 20.3667 |  |  |  |  | Germany | 86.88951 | | a |
|  |  |  |  |  |  |  |  |  |  | |  |
|  |  |  |  |  |  |  |  |  |  | |  |
| 2019 irrigated |  |  |  |  |  |  |  |  |  | |  |
| Samples |  |  |  |  |  |  |  |  |  |  | |
| Anova | | | | | | |  | Tukeys HSD | | | |
|  | Df | Sum Sq | Mean Sq | F value | Pr(>F) |  |  |  | X2019.irr | | groups |
| sample | 1 | 209.8 | 209.783 | 8.5194 | 0.003853 | ** |  | Z71 | 93.23155 | | a |
| Residuals | 236 | 5811.3 | 24.624 |  |  |  |  | Z79 | 91.35378 | | b |
|  |  |  |  |  |  |  |  |  |  | |  |
| Z71 |  |  |  |  |  |  |  |  |  | |  |
| Anova | | | | | | |  | Tukeys HSD | | | |
|  | Df | Sum Sq | Mean Sq | F value | Pr(>F) |  |  |  | X2019.irr | | groups |
| origin | 1 | 6.4 | 6.3723 | 0.2244 | 0.6366 |  |  | Eastern Europe | 93.46199 | | a |
| Residuals | 118 | 3350.8 | 28.3966 |  |  |  |  | Germany | 93.00111 | | a |
|  |  |  |  |  |  |  |  |  |  | |  |
| Z79 |  |  |  |  |  |  |  |  |  | |  |
| Anova | | | | | | |  | Tukeys HSD | | | |
|  | Df | Sum Sq | Mean Sq | F value | Pr(>F) |  |  |  | X2019.irr | | groups |
| origin | 1 | 6.53 | 6.5294 | 0.3095 | 0.5791 |  |  | Germany | 91.58901 | | a |
| Residuals | 116 | 2447.57 | 21.0997 |  |  |  |  | Eastern Europe | 91.11855 | | a |
| Signif. codes: 0 ‘***’ 0.001 ‘**’ 0.01 ‘*’ 0.05 ‘.’ 0.1 ‘ ’ 1 | | | | | | | | | | | |

Table S 4 Rank sum of Relative Leaf Water Content (RLWC) at Z79 across all years. Sorted from highest to lowest rank sum within the groups of origin.

| Variety | Origin | Breed | RLWC Z79 2017 Rainfed | Rank 2017 Rainfed | RLWC Z79 2018 Rainfed | Rank 2018 Rainfed | RLWC Z79 2019 Rainfed | Rank 2019 Rainfed | RLWC Z79 2019 Irrigated | Rank 2019 Irrigated | Rank sum RLWC Z79 |
| --- | --- | --- | --- | --- | --- | --- | --- | --- | --- | --- | --- |
| Rowina | East | Line | 67.26 | 3 | 87.38 | 4 | 85.29 | 30 | 95.70 | 5 | 42 |
| Slava | East | Line | 62.95 | 10 | 89.47 | 1 | 90.77 | 4 | 88.98 | 31 | 46 |
| Numitor | East | Line | 50.57 | 38 | 88.69 | 2 | 92.05 | 2 | 94.57 | 7 | 49 |
| Pajura | East | Line | 65.39 | 6 | 83.85 | 16 | 88.38 | 13 | 92.43 | 17 | 52 |
| Ursita | East | Line | 66.78 | 4 | 82.55 | 22 | 88.94 | 9 | 91.38 | 22 | 57 |
| FGmut 293 | East | Line | 56.48 | 31 | 85.62 | 10 | 90.04 | 7 | 93.24 | 11 | 59 |
| Kuialnik | East | Line | 59.23 | 20 | 80.80 | 31 | 89.15 | 8 | 95.97 | 4 | 63 |
| Unitar | East | Line | 74.29 | 1 | 82.56 | 21 | 84.42 | 33 | 93.88 | 8 | 63 |
| Zagrava | East | Line | 59.96 | 18 | 82.08 | 26 | 86.43 | 25 | 96.65 | 2 | 71 |
| Clasic | East | Line | 63.21 | 8 | 86.83 | 5 | 86.66 | 24 | 86.48 | 36 | 73 |
| Semnal | East | Line | 66.21 | 5 | 81.97 | 28 | 85.01 | 31 | 93.58 | 10 | 74 |
| Meleag | East | Line | 61.79 | 12 | 82.12 | 25 | 90.29 | 6 | 88.52 | 32 | 75 |
| Zisk | East | Line | 48.91 | 40 | 86.19 | 8 | 87.82 | 18 | 92.88 | 14 | 80 |
| Zolotocolosa | East | Line | 67.89 | 2 | 77.59 | 37 | 87.95 | 17 | 90.55 | 25 | 81 |
| Amor | East | Line | 59.51 | 19 | 82.26 | 24 | 87.98 | 16 | 90.13 | 26 | 85 |
| Acord | East | Line | 58.77 | 22 | 84.70 | 13 | 87.49 | 22 | 88.51 | 33 | 90 |
| Savant | East | Line | 56.90 | 29 | 79.34 | 35 | 90.49 | 5 | 90.77 | 24 | 93 |
| Ujinoc | East | Line | 56.40 | 32 | 84.94 | 11 | 87.40 | 23 | 89.45 | 29 | 95 |
| Transitor | East | Line | 63.19 | 9 | 83.26 | 17 | 79.53 | 40 | 89.28 | 30 | 96 |
| Talisman | East | Line | 56.82 | 30 | 82.26 | 23 | 85.86 | 28 | 87.98 | 35 | 116 |
| Hybred | Ger | Hybrid | 61.40 | 13 | 82.00 | 27 | 92.37 | 1 | 97.66 | 1 | 42 |
| JB Asano | Ger | Line | 58.63 | 23 | 84.77 | 12 | 88.85 | 10 | 95.08 | 6 | 51 |
| Apertus | Ger | Line | 62.48 | 11 | 82.76 | 19 | 88.41 | 11 | 91.30 | 23 | 64 |
| Tobak | Ger | Line | 58.10 | 25 | 86.82 | 6 | 88.11 | 15 | 91.57 | 21 | 67 |
| Akteur | Ger | Line | 53.32 | 35 | 84.59 | 14 | 88.40 | 12 | 93.85 | 9 | 70 |
| Patras | Ger | Line | 58.03 | 26 | 86.36 | 7 | 91.65 | 3 | 85.00 | 39 | 75 |
| Genius | Ger | Line | 61.23 | 14 | 83.10 | 18 | 86.27 | 26 | 91.77 | 19 | 77 |
| Kometus | Ger | Line | 63.58 | 7 | 76.66 | 39 | 84.07 | 35 | 96.26 | 3 | 84 |
| Hystar | Ger | Hybrid | 57.51 | 27 | 88.24 | 3 | 82.30 | 38 | 91.75 | 20 | 88 |
| Mulan | Ger | Line | 54.81 | 34 | 84.07 | 15 | 86.03 | 27 | 92.83 | 15 | 91 |
| Elixer | Ger | Line | 53.27 | 36 | 80.92 | 30 | 88.19 | 14 | 93.13 | 12 | 92 |
| Hybery | Ger | Hybrid | 58.47 | 24 | 86.17 | 9 | 84.16 | 34 | 89.71 | 27 | 94 |
| Colonia | Ger | Line | 60.14 | 17 | 80.99 | 29 | 87.81 | 19 | 88.15 | 34 | 99 |
| Kerubino | Ger | Line | 58.86 | 21 | 80.16 | 32 | 82.70 | 37 | 92.49 | 16 | 106 |
| Discus | Ger | Line | 60.91 | 15 | 76.06 | 40 | 81.05 | 39 | 91.77 | 18 | 112 |
| Anapolis | Ger | Line | 57.27 | 28 | 76.90 | 38 | 84.06 | 36 | 93.13 | 13 | 115 |
| Manager | Ger | Line | 60.16 | 16 | 79.47 | 34 | 85.78 | 29 | 85.79 | 38 | 117 |
| Hyfi | Ger | Hybrid | 56.14 | 33 | 79.72 | 33 | 87.68 | 20 | 85.80 | 37 | 123 |
| Impression | Ger | Line | 50.24 | 39 | 79.12 | 36 | 87.57 | 21 | 89.51 | 28 | 124 |
| Rumor | Ger | Line | 52.91 | 37 | 82.66 | 20 | 85.00 | 32 | 84.83 | 40 | 129 |
| EE: Eastern European varieties, Ger: German varieties. | | | | | | | | | | | |

Table S 5 Correlations between the accumulated canopy temperature (CT) and wheat grain yield of wheat groups (all varieties, Eastern European lines, German hybrids, and German lines) under rainfed and irrigated field conditions in Moldova in 2017, 2018, a

| 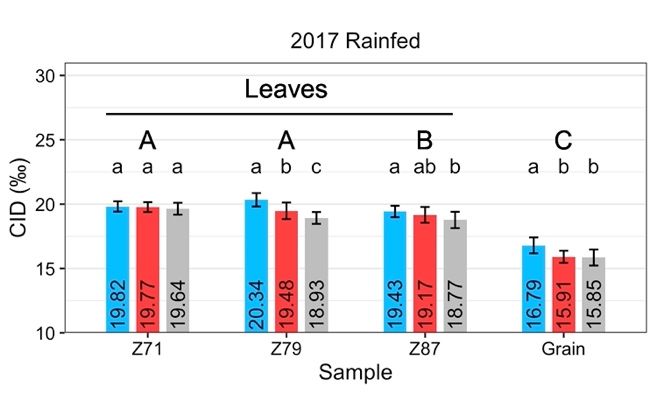 | 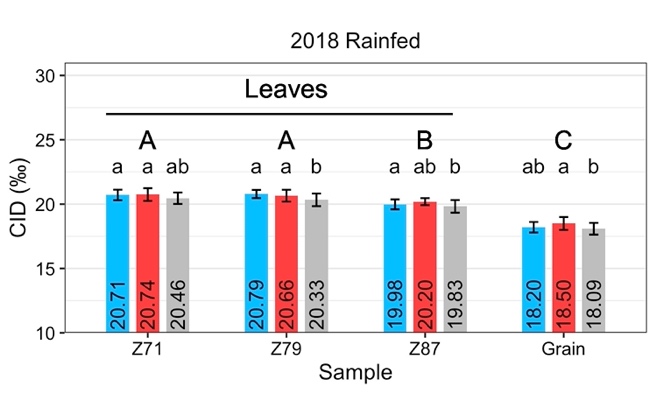 |
| --- | --- |
| 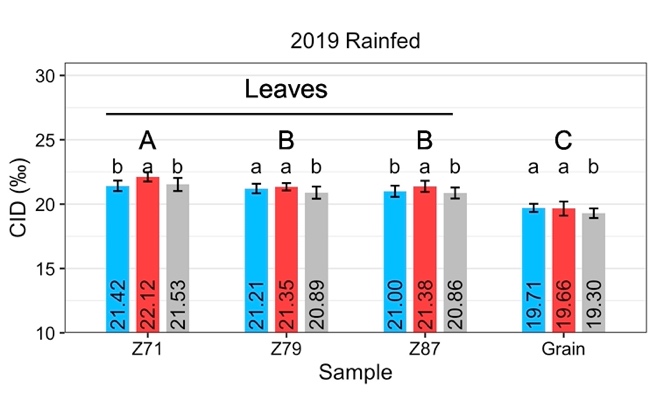 | 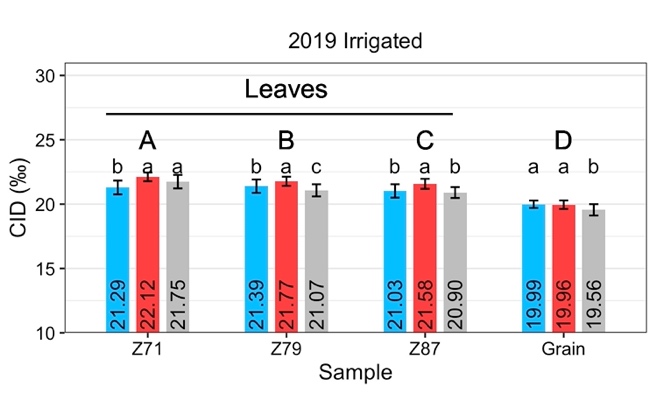 |
| 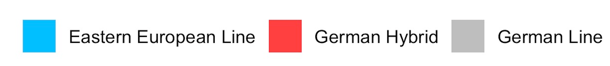 | |

Figure S 3 Carbon isotope discrimination (CID) of wheat leaves at Z71, Z79, and Z87 and grains of Eastern European lines, German lines, and German hybrids under rainfed conditions in 2017, 2018, and 2019 and under irrigated conditions in 2019. The vertical bars indicate the standard deviation of the mean. Capital letters indicate significant differences between the years, small letters indicate significant differences between origins within one year.

Table S 6 Analyses of variance and Tukey-HSD test of CID, dependent on the origin of varieties.

| 2017 |  |  |  |  |  |  |  |  |  |  |
| --- | --- | --- | --- | --- | --- | --- | --- | --- | --- | --- |
| Z71 |  |  |  |  |  |  |  |  |  |  |
| Anova | | | | | | |  | Tukeys HSD | | |
|  | Df | Sum Sq | Mean Sq | F value | Pr(>F) |  |  |  | delta_2017_  promille | groups |
| origin | 1 | 0.4586 | 0.45862 | 2.5329 | 1.16E-01 |  |  | Eastern Europe | 19.82044 | a |
| Residuals | 77 | 13.9418 | 0.18106 |  |  |  |  | Germany | 19.66805 | a |
|  |  |  |  |  |  |  |  |  |  |  |
| Z79 |  |  |  |  |  |  |  |  |  |  |
| Anova | | | | | | |  | Tukeys HSD | | |
|  | Df | Sum Sq | Mean Sq | F value | Pr(>F) |  |  |  | delta_2017_  promille | groups |
| origin | 1 | 48.734 | 48.734 | 173.81 | < 2.2E-16 |  |  | Eastern Europe | 20.33924 | a |
| Residuals | 112 | 31.403 | 0.28 |  |  |  |  | Germany | 19.03138 | b |
|  |  |  |  |  |  |  |  |  |  |  |
| Z87 |  |  |  |  |  |  |  |  |  |  |
| Anova | | | | | | |  | Tukeys HSD | | |
|  | Df | Sum Sq | Mean Sq | F value | Pr(>F) |  |  |  | delta_2017_  promille | groups |
| origin | 1 | 9.851 | 9.8507 | 31.919 | 1.22E-07 | *** |  | Eastern Europe | 19.42981 | a |
| Residuals | 113 | 34.873 | 0.3086 |  |  |  |  | Germany | 18.84426 | b |
|  |  |  |  |  |  |  |  |  |  |  |
| Grains |  |  |  |  |  |  |  |  |  |  |
| Anova | | | | | | |  | Tukeys HSD | | |
|  | Df | Sum Sq | Mean Sq | F value | Pr(>F) |  |  |  | delta_2017_  promille | groups |
| origin | 1 | 26.09 | 26.0901 | 71.169 | 9.52E-14 | *** |  | Eastern Europe | 16.79248 | a |
| Residuals | 118 | 43.258 | 0.3666 |  |  |  |  | Germany | 15.85992 | b |
| Signif. codes: 0 ‘***’ 0.001 ‘**’ 0.01 ‘*’ 0.05 ‘.’ 0.1 ‘ ’ 1 | | | | | | | | | | |
|  |  |  |  |  |  |  |  |  |  |  |
|  |  |  |  |  |  |  |  |  |  |  |
| 2018 |  |  |  |  |  |  |  |  |  |  |
| Z71 |  |  |  |  |  |  |  |  |  |  |
| Anova | | | | | | |  | Tukeys HSD | | |
|  | Df | Sum Sq | Mean Sq | F value | Pr(>F) |  |  |  | delta_2018_  promille | groups |
| origin | 1 | 1.1657 | 1.16571 | 6.1002 | 0.01496 | * |  | Eastern Europe | 20.71227 | a |
| Residuals | 117 | 22.358 | 0.19109 |  |  |  |  | Germany | 20.51432 | b |
|  |  |  |  |  |  |  |  |  |  |  |
| Z79 |  |  |  |  |  |  |  |  |  |  |
| Anova | | | | | | |  | Tukeys HSD | | |
|  | Df | Sum Sq | Mean Sq | F value | Pr(>F) |  |  |  | delta_2018_  promille | groups |
| origin | 1 | 3.5948 | 3.5948 | 21.352 | 1.12E-05 | *** |  | Eastern Europe | 20.78818 | a |
| Residuals | 102 | 17.1725 | 0.1684 |  |  |  |  | Germany | 20.41524 | b |
|  |  |  |  |  |  |  |  |  |  |  |
| Z87 |  |  |  |  |  |  |  |  |  |  |
| Anova |  |  |  |  |  |  |  | Tukeys HSD |  |  |
|  | Df | Sum Sq | Mean Sq | F value | Pr(>F) |  |  |  | delta_2018_  promille | groups |
| origin | 1 | 0.2041 | 0.2041 | 1.0786 | 0.3011 |  |  | Eastern Europe | 19.98307 | a |
| Residuals | 118 | 22.3277 | 0.18922 |  |  |  |  | Germany | 19.90059 | a |
|  |  |  |  |  |  |  |  |  |  |  |
| Grains |  |  |  |  |  |  |  |  |  |  |
| Anova | | | | | | |  | Tukeys HSD | | |
|  | Df | Sum Sq | Mean Sq | F value | Pr(>F) |  |  |  | delta_2018_  promille | groups |
| origin | 1 | 0.0323 | 0.032281 | 0.1586 | 0.6912 |  |  | Eastern Europe | 18.20402 | a |
| Residuals | 118 | 24.0222 | 0.203578 |  |  |  |  | Germany | 18.17121 | a |
| Signif. codes: 0 ‘***’ 0.001 ‘**’ 0.01 ‘*’ 0.05 ‘.’ 0.1 ‘ ’ 1 | | | | | | | | | | |
|  |  |  |  |  |  |  |  |  |  |  |
|  |  |  |  |  |  |  |  |  |  |  |
| 2019 rainfed | | |  |  |  |  |  |  |  |  |
| Z71 |  |  |  |  |  |  |  |  |  |  |
| Anova | | | | | | |  | Tukeys HSD | | |
|  | Df | Sum Sq | Mean Sq | F value | Pr(>F) |  |  |  | delta_2019dry_  promille | groups |
| origin | 1 | 1.5163 | 1.51627 | 6.6982 | 0.01086 | * |  | Germany | 21.64581 | a |
| Residuals | 118 | 26.7117 | 0.22637 |  |  |  |  |  | 21.42099 | b |
|  |  |  |  |  |  |  |  |  |  |  |
| Z79 |  |  |  |  |  |  |  |  |  |  |
| Anova | | | | | | |  | Tukeys HSD | | |
|  | Df | Sum Sq | Mean Sq | F value | Pr(>F) |  |  |  | delta_2019dry_  promille | groups |
| origin | 1 | 1.5313 | 1.53127 | 8.4393 | 0.004398 | ** |  | Eastern Europe | 21.21326 | a |
| Residuals | 116 | 21.0477 | 0.18145 |  |  |  |  | Germany | 20.98539 | b |
|  |  |  |  |  |  |  |  |  |  |  |
| Z87 |  |  |  |  |  |  |  |  |  |  |
| Anova | | | | | | |  | Tukeys HSD | | |
|  | Df | Sum Sq | Mean Sq | F value | Pr(>F) |  |  |  | delta_2019dry_  promille | groups |
| origin | 1 | 0.0273 | 0.027277 | 0.1343 | 0.7147 |  |  | Eastern Europe | 20.99714 | a |
| Residuals | 118 | 23.9719 | 0.203152 |  |  |  |  | Germany | 20.96698 | a |
|  |  |  |  |  |  |  |  |  |  |  |
| Grains |  |  |  |  |  |  |  |  |  |  |
| Anova | | | | | | |  | Tukeys HSD | | |
|  | Df | Sum Sq | Mean Sq | F value | Pr(>F) |  |  |  | delta_2019dry_  promille | groups |
| origin | 1 | 3.5057 | 3.5057 | 24.271 | 2.76E-06 | *** |  | Eastern Europe | 19.70933 | a |
| Residuals | 118 | 17.0441 | 0.1444 |  |  |  |  | Germany | 19.36748 | b |
| Signif. codes: 0 ‘***’ 0.001 ‘**’ 0.01 ‘*’ 0.05 ‘.’ 0.1 ‘ ’ 1 | | | | | | | | | | |
|  |  |  |  |  |  |  |  |  |  |  |
|  |  |  |  |  |  |  |  |  |  |  |
| 2019 irrigated | | | |  |  |  |  |  |  |  |
| Z71 |  |  |  |  |  |  |  |  |  |  |
| Anova | | | | | | |  | Tukeys HSD | | |
|  | Df | Sum Sq | Mean Sq | F value | Pr(>F) |  |  |  | delta_2019irr_  promille | groups |
| origin | 1 | 8.41 | 8.4104 | 30.535 | 1.99E-07 | *** |  | Germany | 21.821 | a |
| Residuals | 118 | 32.501 | 0.2754 |  |  |  |  | Eastern Europe | 21.29153 | b |
|  |  |  |  |  |  |  |  |  |  |  |
| Z79 |  |  |  |  |  |  |  |  |  |  |
| Anova | | | | | | |  | Tukeys HSD | | |
|  | Df | Sum Sq | Mean Sq | F value | Pr(>F) |  |  |  | delta_2019irr_  promille | groups |
| origin | 1 | 0.954 | 0.95411 | 3.4442 | 0.06601 | . |  | Eastern Europe | 21.39453 | a |
| Residuals | 116 | 32.134 | 0.27702 |  |  |  |  | Germany | 21.21469 | a |
|  |  |  |  |  |  |  |  |  |  |  |
| Grain |  |  |  |  |  |  |  |  |  |  |
| Anova | | | | | | |  | Tukeys HSD | | |
|  | Df | Sum Sq | Mean Sq | F value | Pr(>F) |  |  |  | delta_2019irr_  promille | groups |
| origin | 1 | 3.6339 | 3.6339 | 25.919 | 1.39E-06 | *** |  | Eastern Europe | 19.99156 | a |
| Residuals | 116 | 16.2637 | 0.1402 |  |  |  |  | Germany | 19.64054 | b |
| Signif. codes: 0 ‘***’ 0.001 ‘**’ 0.01 ‘*’ 0.05 ‘.’ 0.1 ‘ ’ 1 | | | | | | | | | | |

Table S 7 Rank sum of CID of grains across all years. Sorted from highest to lowest rank sum within the groups of origin.

| Variety | Origin | Breed | CID Grain 2017 Rainfed | Rank 2017 Rainfed | CID Grain 2018 Rainfed | Rank 2018 Rainfed | CID Grain 2019 Rainfed | Rank 2019 Rainfed | CID Grain 2019 Irrigated | Rank 2019 Irrigated | Rank sum CID |
| --- | --- | --- | --- | --- | --- | --- | --- | --- | --- | --- | --- |
| Ursita | East | Line | 17.21 | 1 | 18.45 | 7 | 20.03 | 4 | 20.35 | 1 | 13 |
| Talisman | East | Line | 16.77 | 12 | 18.80 | 3 | 19.92 | 6 | 20.25 | 4 | 25 |
| Unitar | East | Line | 17.20 | 2 | 18.55 | 5 | 19.50 | 21 | 20.16 | 7 | 35 |
| Zolotocolosa | East | Line | 16.95 | 7 | 18.52 | 6 | 19.75 | 11 | 20.09 | 11 | 35 |
| Numitor | East | Line | 16.94 | 8 | 18.29 | 14 | 19.78 | 10 | 20.04 | 14 | 46 |
| Acord | East | Line | 16.92 | 10 | 18.24 | 18 | 19.88 | 7 | 19.95 | 16 | 51 |
| FGmut 293 | East | Line | 17.04 | 3 | 18.34 | 11 | 19.68 | 16 | 19.83 | 21 | 51 |
| Meleag | East | Line | 16.41 | 18 | 17.88 | 25 | 19.86 | 8 | 20.32 | 2 | 53 |
| Clasic | East | Line | 16.24 | 20 | 18.25 | 15 | 19.73 | 12 | 20.22 | 6 | 53 |
| Savant | East | Line | 16.48 | 16 | 18.13 | 20 | 19.99 | 5 | 20.08 | 13 | 54 |
| Amor | East | Line | 16.19 | 21 | 18.07 | 22 | 20.06 | 3 | 20.14 | 10 | 56 |
| Transitor | East | Line | 17.03 | 5 | 18.24 | 16 | 19.62 | 17 | 19.91 | 18 | 56 |
| Zagrava | East | Line | 17.04 | 4 | 18.24 | 17 | 19.68 | 13 | 19.79 | 22 | 56 |
| Rowina | East | Line | 16.51 | 15 | 18.10 | 21 | 19.81 | 9 | 19.92 | 17 | 62 |
| Kuialnik | East | Line | 16.86 | 11 | 18.37 | 9 | 19.54 | 20 | 19.77 | 23 | 63 |
| Zisk | East | Line | 16.92 | 9 | 18.22 | 19 | 19.47 | 22 | 20.03 | 15 | 65 |
| Semnal | East | Line | 16.57 | 14 | 17.60 | 30 | 19.27 | 25 | 20.08 | 12 | 81 |
| Pajura | East | Line | 16.68 | 13 | 17.70 | 29 | 19.59 | 18 | 19.64 | 25 | 85 |
| Elixer | Ger | Line | 16.25 | 19 | 18.59 | 4 | 20.10 | 2 | 20.14 | 8 | 33 |
| Hybery | Ger | Hybrid | 15.81 | 25 | 18.39 | 8 | 20.36 | 1 | 20.30 | 3 | 37 |
| Hyfi | Ger | Hybrid | 16.04 | 22 | 18.86 | 2 | 19.68 | 15 | 20.14 | 9 | 48 |
| Anapolis | Ger | Line | 16.96 | 6 | 18.35 | 10 | 19.57 | 19 | 19.90 | 19 | 54 |
| Hystar | Ger | Hybrid | 16.43 | 17 | 18.87 | 1 | 19.44 | 23 | 19.86 | 20 | 61 |
| Mulan | Ger | Line | 15.80 | 26 | 17.95 | 23 | 19.68 | 14 | 20.24 | 5 | 68 |
| Colonia | Ger | Line | 15.79 | 27 | 18.32 | 13 | 19.23 | 26 | 19.68 | 24 | 90 |
| Discus | Ger | Line | 15.62 | 28 | 18.34 | 12 | 19.21 | 28 | 19.51 | 29 | 97 |
| Impression | Ger | Line | 14.96 | 32 | 17.94 | 24 | 19.30 | 24 | 19.54 | 27 | 107 |
| Patras | Ger | Line | 15.97 | 23 | 17.76 | 27 | 19.13 | 30 | 19.45 | 30 | 110 |
| Hybred | Ger | Hybrid | 15.36 | 30 | 17.88 | 26 | 19.14 | 29 | 19.53 | 28 | 113 |
| Manager | Ger | Line | 15.49 | 29 | 17.54 | 32 | 19.21 | 27 | 19.60 | 26 | 114 |
| Genius | Ger | Line | 15.91 | 24 | 17.70 | 28 | 18.98 | 31 | 19.26 | 31 | 114 |
| Kerubino | Ger | Line | 15.19 | 31 | 17.55 | 31 | 18.97 | 32 | 19.24 | 32 | 126 |
| EE: Eastern European varieties, Ger: German varieties. | | | | | | | | | | | |
